# Kaposi’s sarcoma-associated herpesvirus ORF57 protein protects viral transcripts from specific nuclear RNA decay pathways by preventing hMTR4 recruitment

**DOI:** 10.1371/journal.ppat.1007596

**Published:** 2019-02-20

**Authors:** Julio C. Ruiz, Olga V. Hunter, Nicholas K. Conrad

**Affiliations:** Department of Microbiology, University of Texas Southwestern Medical Center, Dallas, Texas; University of Utah, UNITED STATES

## Abstract

Nuclear RNAs are subject to a number of RNA decay pathways that serve quality control and regulatory functions. As a result, any virus that expresses its genes in the nucleus must have evolved mechanisms that avoid these pathways, but the how viruses evade nuclear RNA decay remains largely unknown. The multifunctional Kaposi’s sarcoma-associated herpesvirus (KSHV) ORF57 (Mta) protein is required for the nuclear stability of viral transcripts. In the absence of ORF57, we show that viral transcripts are subject to degradation by two specific nuclear RNA decay pathways, PABPN1 and PAPα/γ-mediated RNA decay (PPD) in which decay factors are recruited through poly(A) tails, and an ARS2-mediated RNA decay pathway dependent on the 5ʹ RNA cap. In transcription pulse chase assays, ORF57 appears to act primarily by inhibiting the ARS2-mediated RNA decay pathway. In the context of viral infection in cultured cells, inactivation of both decay pathways by RNAi is necessary for the restoration of ORF57-dependent viral genes produced from an ORF57-null bacmid. Mechanistically, we demonstrate that ORF57 protects viral transcripts by preventing the recruitment of the exosome co-factor hMTR4. In addition, our data suggest that ORF57 recruitment of ALYREF inhibits hMTR4 association with some viral RNAs, whereas other KSHV transcripts are stabilized by ORF57 in an ALYREF-independent fashion. In conclusion, our studies show that KSHV RNAs are subject to nuclear degradation by two specific host pathways, PPD and ARS2-mediated decay, and ORF57 protects viral transcripts from decay by inhibiting hMTR4 recruitment.

## Author summary

Eukaryotic cells contain numerous nuclear RNA quality control (QC) systems that ensure transcriptome fidelity by detecting and degrading aberrant RNAs. Some viral RNAs are also predicted to be degraded by these RNA QC systems, so viruses have evolved mechanisms that counter host RNA QC pathways. Previous studies showed that the Kaposi’s sarcoma-associated herpesvirus (KSHV) expresses the ORF57 protein to protect its RNAs from nuclear decay. However, neither the specific host pathways that degrade KSHV RNAs nor the mechanisms describing ORF57 protection of viral RNAs were known. Our data suggest that ORF57 protects viral RNAs from two different nuclear RNA QC pathways, PABPN1 and PAPα/γ-mediated RNA decay (PPD) and an ARS2-mediated RNA decay pathway. Mechanistically, we show that ORF57 binds directly to viral RNAs and prevents the recruitment of hMTR4, a cellular factor whose function is to recruit the exosome, the complex responsible for RNA decay, to the transcript. We conclude that by preventing hMTR4 recruitment, ORF57 protects viral RNAs from degradation resulting in robust expression of viral genes.

## Introduction

The Kaposi’s sarcoma-associated herpesvirus (KSHV) is a nuclear double-stranded DNA virus that belongs to the gammaherpesvirus family. It infects human B lymphocytes and endothelial cells and causes Kaposi’s sarcoma (KS) as well as the lymphoproliferative disorders primary effusion lymphoma (PEL) and some cases of multicentric Castleman’s disease (MCD) [1–4]. The KSHV life cycle consists of latent and lytic phases of infection. During the latent phase, the viral genome resides in the host nucleus as a circular episome and only a few viral genes are expressed. Upon reactivation to the lytic phase, a well-regulated cascade of gene expression is initiated by the transactivator ORF50 (Rta) that results in the production of infectious virus [5–8]. Similar to other herpesviruses, the KSHV genome is nuclear, and it expresses its genes using the host cells transcription, RNA processing and translation machinery. Consequently, viral transcripts, like their host counterparts, are subject to RNA quality control (RNA QC) pathways that degrade improperly processed, aberrant RNAs [9–13].

In eukaryotes, RNA QC systems play an essential role in RNA metabolism [9–13]. Messenger RNA biogenesis requires the pre-mRNA to undergo several maturation processes within the nucleus. A nascent mRNA is capped, spliced, polyadenylated and exported to the cytoplasm where it is translated. Transcripts that fail to proceed through these maturation processes are retained in the nucleus and degraded by RNA QC pathways. These same pathways are important for degrading functional and nonfunctional nuclear noncoding RNAs (ncRNAs). For example, promoter upstream transcripts (PROMPTs; also called uaRNAs) are polyadenylated, non-coding RNAs with no or few introns that are made from bi-directional protein-coding promoters [14–16]. RNA QC pathways rapidly degrade PROMPTs in the nucleus [17–19]. Otherwise, these RNAs could be translated into potentially toxic proteins or compete with mRNAs for the translation machinery [20]. Some processed noncoding miRNA and noncoding snoRNA host genes (ncSNHGs) are also degraded in the nucleus by RNA QC pathways [17, 18, 21, 22]. Furthermore, these decay pathways can control the levels of functional noncoding nuclear RNAs [18, 21, 22]. Thus, RNA QC pathways target a variety of nuclear transcripts, so both host and viral RNAs must evade them in order to be expressed.

The PABPN1 and PAPα/γ-mediated RNA decay (PPD) pathway is an RNA decay pathway that degrades polyadenylated nuclear transcripts [18, 21, 22]. In this pathway, the nuclear poly(A)-binding protein (PABPN1) binds to its substrate’s poly(A) tail and promotes further poly(A) tail extension by the canonical poly(A) polymerases, PAPα and PAPγ (PAPOLA and PAPOLG). This “hyperadenylated” RNA is then degraded exonucleolytically in a 3′ to 5′ fashion by the nuclear exosome [22]. The mechanism of coupling between hyperadenylation and exosome-mediated decay remains unknown. However, we speculated that the generation of a nascent poly(A) tail creates a competition between poly(A)-binding proteins and the exosome [22]. Recently, two groups described the poly(A) tail exosome targeting (PAXT) connection (alternatively called the polysome protector complex, PPC) in which the zinc finger protein ZFC3H1 links the exosome cofactor hMTR4 to PABPN1, thus recruiting the exosome to polyadenylated RNAs to be degraded [19, 20]. PPD/PAXT targets poorly exported polyadenylated RNAs as well as PROMPTs, ncSNHGs, lncRNAs and pri-miRNAs [18–22]. Thus, PPD is an important nuclear RNA decay pathway that eliminates inefficiently processed RNAs and modulates the levels of non-coding RNAs.

Nuclear transcripts can alternatively be eliminated by a pathway called the CBCN-mediated decay pathway that involves two complexes: the cap-binding complex (CBC) and the nuclear exosome targeting (NEXT) complex [17]. The CBC is a heterodimer of CBP20 and CBP80 proteins that associates co-transcriptionally with the 5′-methyl guanosine cap of RNA polymerase II transcripts. CBC recruits multiple distinct factors to mediate several steps of RNA biogenesis [23]. To mediate RNA degradation, the CBC associates with ARS2 (arsenite-resistance protein 2) which in turn recruits the NEXT complex, consisting of ZCCHC8, RBM7 and hMTR4 [17, 24, 25] to form the CBCN complex. The NEXT complex serves as a platform that connects the nuclear exosome with the CBC at the 5′-end of transcripts to be degraded [24]. In this complex, RBM7 binds directly to the RNA target and the zinc finger protein ZCCHC8 connects RBM7 to hMTR4, which in turn recruits the nuclear exosome to its substrates [26]. Furthermore, the CBC-ARS2 (CBCA) complex, a CBCN subcomplex, links the 5′-end cap to 3′-end maturation and triggers the termination of several RNA families including PROMPTs [27]. Thus, CBCN prevents the accumulation of potentially deleterious transcriptional by products.

KSHV transcripts resemble those of their host in that they are capped and polyadenylated. However, 70% of KSHV genes are intronless while the majority of human protein-coding genes contain multiple introns [28, 29]. Most steps in RNA biogenesis including transcription initiation and elongation, capping, 3′-end processing, mRNA export and translation are mechanistically coupled in such a way that splicing or the presence of an intron facilitates efficient gene expression [30–36]. Moreover, the presence of a 5ʹ splice site appears to be part of the mechanism that cells use to distinguish between PROMPTs and stable mRNAs [37, 38]. Due to their shorter gene length and lack of introns, KSHV transcripts are similar to PROMPTs, so they are predicted to succumb to host RNA QC pathways.

In order to avoid transcript decay in the nucleus, KSHV has evolved mechanisms that allow efficient expression of its genes in the absence of splicing. The KSHV ORF57 (Mta) protein is a post-transcriptional regulator of gene expression that is essential for virus replication [39–42]. Homologs are found in all herpesvirus but not in any known host organism [43–45]. ORF57 is a multifunctional protein that has been implicated in nearly every step of viral RNA biogenesis, and it allows efficient viral gene expression in the absence of splicing [46–52]. Importantly, ORF57 increases the nuclear stability of viral RNAs. For example, ORF57 enhances the expression of PAN RNA, an intronless, nuclear non-coding transcript that accumulates at high levels during the lytic phase of infection [40, 53–56]. PAN RNA accumulation depends on a 79-nucleotide (nt) stability element called ENE that interacts in cis with the poly(A) tail and sequesters it from exonucleases [57–59]. Deletion of the ENE renders PAN RNA unstable and is rapidly degraded. ORF57 directly binds to and stabilizes the unstable form of PAN RNA (PANΔENE) as well as WT PAN RNA [60–63]. Furthermore, ORF57 recruits the RNA export factor ALYREF to promote PAN RNA nuclear stabilization in an export-independent manner [64]. In addition to PAN RNA, ORF57 posttranscriptionally functions to promote the accumulation of specific viral mRNAs [41, 56, 65–69]. Overall, these observations imply that ORF57 enhances KSHV gene expression by protecting viral transcripts from host-mediated RNA decay pathways. However, neither the RNA decay pathway(s) inhibited by ORF57 nor the mechanism(s) by which ORF57 protects viral RNAs from degradation is known.

Here we used RNAi to target host nuclear decay pathways and monitored their contributions to the degradation of PANΔENE. Depletion of cellular factors involved in PPD and depletion of ARS2 stabilized the transcript, but knockdown of NEXT components had no effect on decay. In transcription pulse-chase assays, ORF57 protects PANΔENE from ARS2-mediated decay, but it is unclear whether it protects PANΔENE from PPD. In the context of viral infection in cultured cells, inactivation of both decay pathways restored the expression of a subset of ORF57-dependent viral genes produced from an ORF57-null bacmid. Thus, our data suggest that ORF57-mediated protection of viral RNAs from these nuclear decay pathways is important for viral replication. Mechanistically, we show that ORF57 inhibits recruitment of the exosome co-factor hMTR4 to viral RNAs. In addition, our data suggest that ORF57 promotes the recruitment of ALYREF to some viral RNAs to increase the stability of the viral transcript presumably by impeding the interaction between ARS2 and hMTR4. We conclude that ORF57 contributes to KSHV gene expression by hampering the activity of host-mediated nuclear RNA QC pathways.

## Results

### Both PPD inactivation and ORF57 expression are required for optimal PANΔENE stabilization in pulse-chase reporter assays

**Fig 1:**
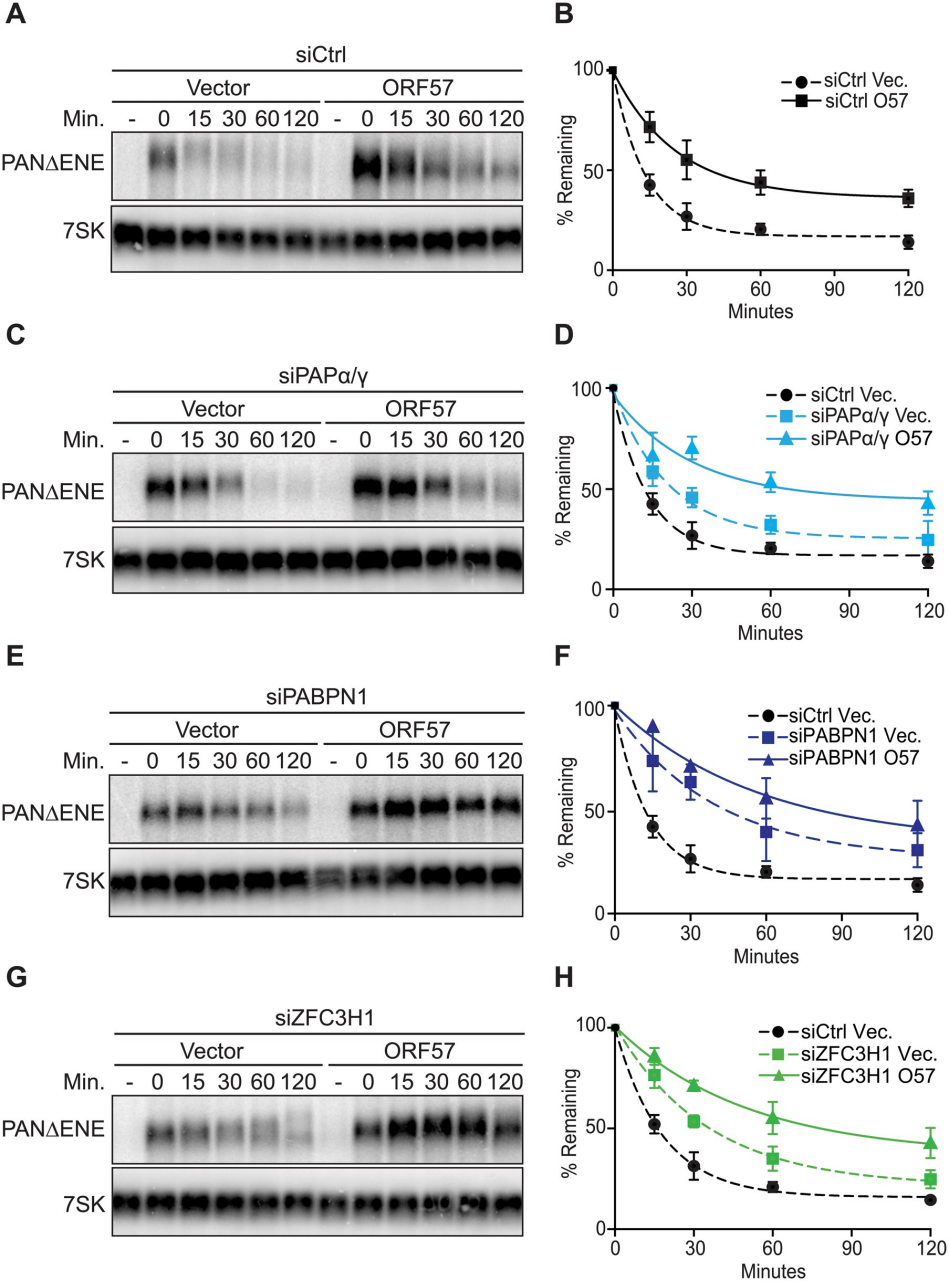
ORF57 expression stabilizes PANΔENE in pulse-chase assays upon PPD inactivation. (A, C, E & G) Representative northern blots of transcription pulse-chase assay in 293A-TOA cells transfected with an empty vector or ORF57 expression plasmid and with a non-targeting control siRNA (A) or a two-siRNA pool targeting PAPα/γ (C), PABPN1 (E), or ZFC3H1 (G). 7SK serves as loading control. The “-” lane was harvested prior to dox removal and the time 0 sample was taken two hours after dox removal immediately prior to re-introduction of dox. (B, D, F & H) Decays curves of biological replicates of the transcription pulse-chase assays; each point is a mean value with standard deviation (n = 3). Quantification was performed by normalizing PANΔENE values to 7SK. The t = 0 sample was set to 100% and other values were calculated relative to this sample. For each graph in Figs 1 and 2, the siCtrl/Vector data are re-plotted as a reference.

To identify host-mediated nuclear RNA decay pathways that target KSHV viral transcripts for degradation and to determine whether ORF57 acts on these pathways, we used a well-defined transcription pulse-chase assay [22, 57, 61, 70]. In this assay, 293A-TOA cells are transfected with a plasmid expressing PANΔENE under the control of the tetracycline-responsive promoter. Transcription from this promoter is induced by removing doxycycline (dox) for two hours, then repressed by readdition of dox. Initially, we confirmed our previous results that PANΔENE is more stable in the presence of ORF57 (Fig 1A and 1B) [61]. This observation reaffirms the idea that ORF57 protects viral transcripts from a yet to be identified cellular RNA decay pathway.

PPD degrades polyadenylated RNAs with few or no introns, at least partially due to their inefficient export [18]. Moreover, inactivation of the PPD protects PANΔENE from degradation [22], so we hypothesized that ORF57 protects viral transcripts from decay by inhibiting PPD. To test this idea, we inactivated PPD by depleting cells of PABPN1, PAPα and PAPγ, or ZFC3H1 and monitored PANΔENE stability in the presence or absence of ORF57. Efficiency of knockdown was validated by western blot, qRT-PCR and/or loss of function assays (S1 Fig). From the pulse-chase assay, we expected one of two possible outcomes. If ORF57 protects PANΔENE from degradation by inhibiting PPD, then PANΔENE should be stabilized to the same extent in the presence or in absence of ORF57 upon PPD inactivation. In contrast, if ORF57 inhibits a separate decay pathway in addition to (or instead of) PPD, then PANΔENE should be further stabilized in the presence of ORF57. As expected, PAPα/γ and PABPN1 depletion increased PANΔENE stability in the absence of ORF57 (Fig 1C–1F) [22]. Similarly, depletion of ZFC3H1, a recently identified member of this decay pathway [19, 20], increased PANΔENE stability (Fig 1G and 1H). Importantly, ORF57 expression resulted in a more robust stabilization of PANΔENE in each case (Fig 1C–1H). These data demonstrate that PPD targets viral PANΔENE RNA, but suggest that another decay pathway contributes to its degradation. Moreover, ORF57 appears to protect PANΔENE from this distinct cellular RNA decay pathway. Because we cannot perform knockouts of these essential genes, it is alternatively possible that the more robust stabilization by ORF57 results from its inhibiting residual PPD activity that remains after knockdown. Therefore, we cannot definitively exclude a role for ORF57 in protecting PANΔENE from PPD.

### ORF57 inhibits an ARS2-dependent, NEXT-independent decay pathway in pulse-chase reporter assays

**Fig 2:**
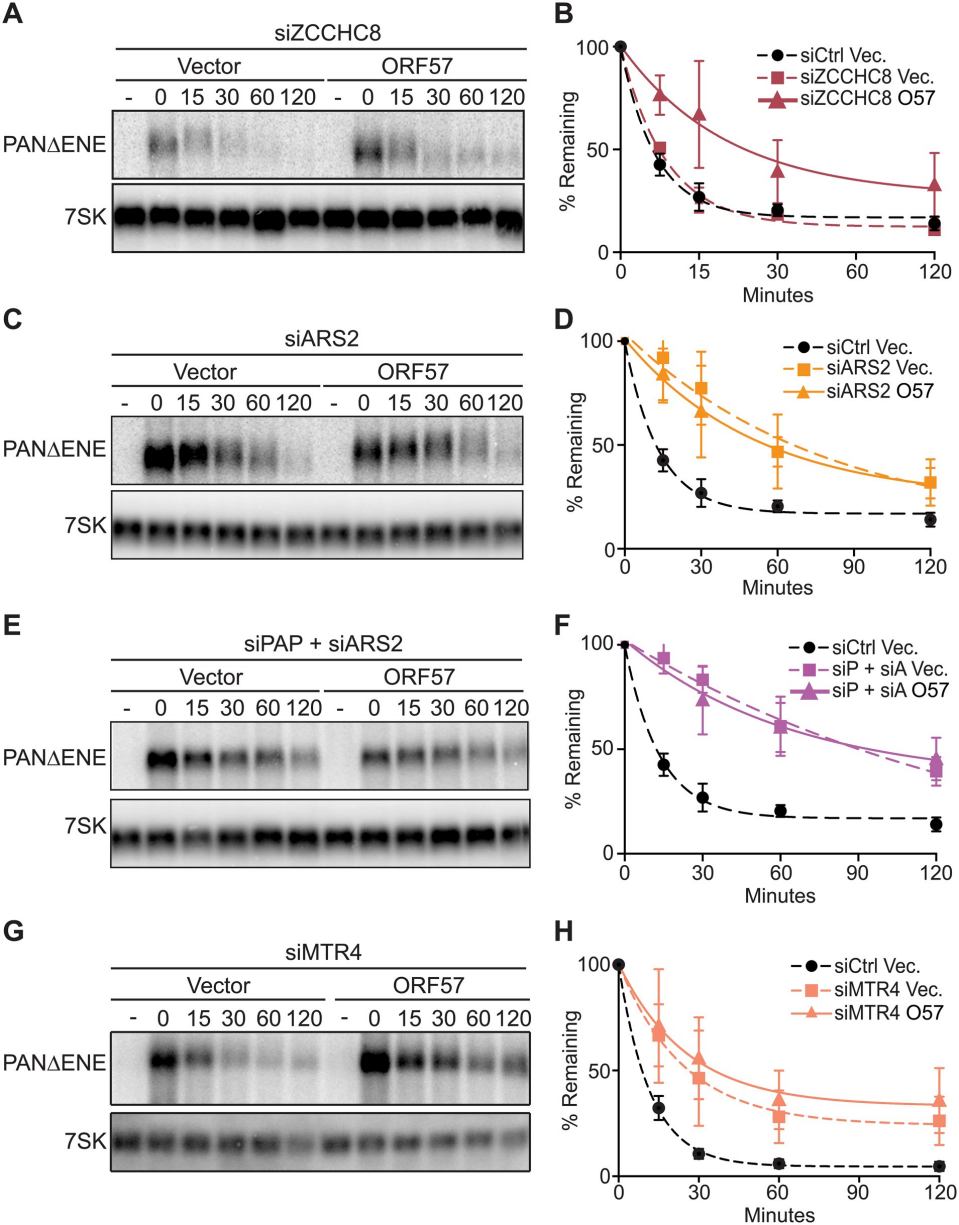
ORF57 acts on an ARS2-mediated RNA decay pathway. (A, C, E & G) Representative northern blots of transcription pulse-chase assay in cells expressing an empty vector or ORF57 and transfected with a two-siRNA pool targeting ZCCHCH8 (A), ARS2 (C), PAPα/γ and ARS2 combined (E) or hMTR4 (G). 7SK serves as loading control. (B, D, F & H) Decay curves of biological replicates of the transcription pulse-chase assays (n = 3). See Fig 1 for additional details.

We next investigated the role of the CBCN-mediated nuclear RNA decay pathway in PANΔENE decay and ORF57 stabilization. To do so, we monitored PANΔENE stability upon CBCN inactivation by knocking down the NEXT components RBM7 or ZCCHC8 in the presence or in the absence of ORF57. Both RBM7 and ZCCHC8 were efficiently depleted at the mRNA level (S1B Fig) and functional knockdown was confirmed by observed increased RNA levels of NEXT-targeted PROMPTs (S1D Fig). Depletion of ZCCHC8 or RBM7, did not stabilize PANΔENE in the absence of ORF57 (Fig 2A and 2B and S2A and S2B Fig), suggesting that NEXT is not involved in PANΔENE degradation. We also tested a potential role for the CBC-binding protein ARS2. In contrast to the NEXT depletion experiments, PANΔENE RNA was stabilized when ARS2 was depleted suggesting that an ARS2-dependent decay pathway targets PANΔENE RNA for degradation (S1A Fig and Fig 2C and 2D). Notably, ORF57 expression in the context of ARS2 depletion did not further stabilize PANΔENE, suggesting that ORF57 protects PANΔENE from decay by inhibiting an ARS2-dependent, but NEXT-independent decay pathway (Fig 2C and 2D).

Our data suggest that both PPD and an ARS2-dependent decay pathway target PANΔENE for degradation but ORF57 preferentially inhibits the latter. Therefore, we tested whether these two pathways are redundant or they are part of the same pathway. We co-depleted PAPα/γ and ARS2 and monitored PANΔENE stability by northern blot. Inactivation of both decay pathways in the absence of ORF57 stabilized PANΔENE to a greater extent than PAPα/γ or ARS2 alone (compare Fig 2E and 2F with 2C and 2D and Fig 1). Furthermore, co-expression of ORF57 did not increase PANΔENE stability any further (Fig 2E and 2F). These data suggest that PPD and an ARS2-dependent decay pathway independently target PANΔENE for degradation and that ORF57 inhibits the latter in pulse-chase assays.

The nuclear exosome requires cofactors to carry out its functions including the RNA helicase hMTR4 that has been implicated to be part of both CBCN and PPD [17, 19, 20, 71, 72]. Upon hMTR4 knockdown (S1A Fig), PANΔENE was stabilized (Fig 2G and 2H). Importantly, co-expression of ORF57 did not have a significant additive effect on PANΔENE stability consistent with the inactivation of both pathways by hMTR4 (Fig 2G and 2H). Taken together, our data suggest that PPD and an ARS2-dependent but NEXT-independent pathway targets PANΔENE for degradation. Moreover, our results suggest that ORF57 protects PANΔENE from the ARS2-dependent decay.

### Generation and characterization of iSLK-ΔORF57 cells

**Fig 3:**
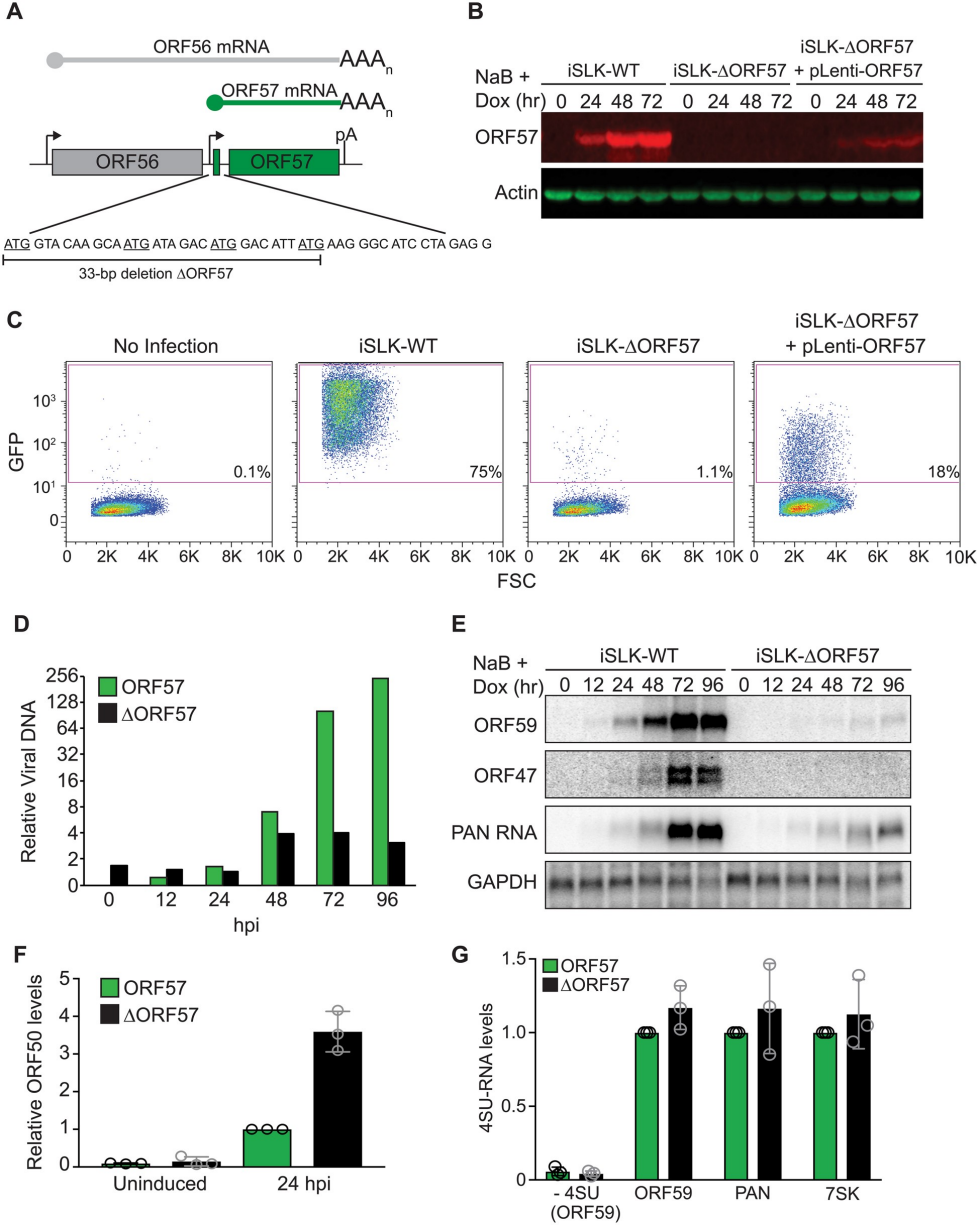
Generation and characterization of iSLK-ΔORF57 cells. (A) Schematic diagram of ORF57 locus and deletion strategy. ORF56 (gray) and ORF57 (green) share a poly(A) signal. A 33-bp deletion containing the ORF57 initiating ATG and three additional in-frame ATG codons was made to minimize effects on ORF56 expression. (B) Quantitative western blot showing ORF57 protein from induced iSLK-WT, iSLK-ΔORF57 or iSLK-ΔORF57 cells transduced with lentivirus expressing ORF57. Actin serves as loading control. (C) Flow cytometry analysis of HEK293 cells infected with supernatants from indicated cell lines. The x-axis shows forward scatter and the y-axis indicates GFP. Percentage of GFP positives is shown. (D) Relative viral DNA levels in iSLK-WT (green) and iSLK-ΔORF57 (black) cells after induction. Values were calculated relative to WT 0 hours post induction (hpi). (E) Time course and northern blot of ORF59, ORF47 and PAN RNA mRNA from iSLK-WT and iSLK-ΔORF57 cells. (F) Relative ORF50 mRNA levels in iSLK-WT (green) and iSLK-ΔORF57 (black) determined by qRT-PCR either not induced or 24 hours post lytic reactivation. Values were first normalized to β-actin and calculated relative to WT (n = 3). In this and all figures, the open circles represent values from each biological replicate. (G) Ten-minute 4SU pulse labelling of iSLK-WT (green) and iSLK-ΔORF57 (black) cells. 4SU-containing ORF59, PAN RNA, and 7SK levels were measured by qRT-PCR after selection. Quantification was done by setting the value of WT samples to 1 for each transcript (i.e. ORF59, PAN RNA and 7SK). ΔORF57 values were calculated relative to the corresponding WT for each of three biological replicates. 7SK shows that there is no change between WT and ΔORF57 samples. The “- 4SU (ORF59)” samples used RNA from the indicated reactivated cell lines but were not treated with 4SU. These samples show background of the assay and were calculated relative to ORF59 WT. Values are average and error bars are standard deviations (n = 3).

To expand our studies of the role of ORF57 in nuclear decay to viral infection, we generated an ORF57 knockout using the KSHV infectious clone BAC16 [73]. The intron-containing ORF57 gene shares a poly(A) signal with the upstream ORF56 gene (Fig 3A), so complete deletion of ORF57 could potentially alter the expression of ORF56. To circumvent this issue, we made a 33-bp deletion that includes ORF57 initiating ATG and three additional in-frame ATG codons (Fig 3A). We transfected iSLK cells with either BAC16 (WT) or ΔORF57 bacmid to generate iSLK-WT and iSLK-ΔORF57 cells, respectively. iSLK cells encode a dox-inducible Rta (ORF50) gene, a master transactivator that is necessary and sufficient to promote viral lytic reactivation [74]. Viral reactivation with dox alone is modest, so we treat iSLK cells with dox and the histone deacetylase inhibitor sodium butyrate (NaB) to promote robust reactivation. In iSLK-WT cells, ORF57 is expressed after induction, but undetectable in ΔORF57 cells (Fig 3B). As ORF57 is essential for virus replication [40, 55], we confirmed that iSLK-ΔORF57 cells are unable to produce infectious virions. We collected media from iSLK-WT and iSLK-ΔORF57 cells 96 hours after lytic reactivation and used it to infect HEK293 cells. Two days later, infected cells were analyzed by flow cytometry to detect the GFP expressed by BAC16. Most of the HEK293 cells infected with media collected from iSLK-WT cells were GFP positive, but almost no cells were GFP positive after infection with iSLK-ΔORF57 media (Fig 3C). Importantly, we complemented iSLK-ΔORF57 cells in trans by transducing them with a lentivirus expressing wild type ORF57 gene driven by its own promoter. As expected, transduction of ORF57 led to an ~18-fold increase in infectious virions (Fig 3C, last panel). The lack of complementation to wild type levels is presumably due to lower expression of ORF57 in these cells (Fig 3B). Further characterization of iSLK-ΔORF57 cells revealed that the virus is unable to replicate its genome properly upon lytic reactivation (Fig 3D) and that the expression of several known ORF57-dependent viral RNAs is severely impaired in reactivated iSLK-ΔORF57 cells (Fig 3E). It is noteworthy that during latency (0 hpi) the virus genome copy number is slightly higher in iSLK-ΔORF57 cells (Fig 3D), so deficiencies in virus production or gene expression are not due to lower DNA copy number. In addition, these deficiencies in gene expression and virus production are not due to compromised expression of ORF50 in iSLK-ΔORF57 cells as ORF50 is expressed even at higher levels than in iSLK-WT cells (Fig 3F). Taken together, these results validate our recombinant ORF57-null bacmid for studies of ORF57 mechanisms.

ORF57 has been implicated in both transcriptional and posttranscriptional regulation, either of which could be affected in the context viral infection. Because much our work here focuses on two well characterized ORF57 target genes, PAN RNA and ORF59 mRNA, we tested whether the reduced expression of these transcripts in iSLK-ΔORF57 cells was due to a transcriptional or a post-transcriptional defect. To do so, we treated cells for 10 minutes with 4-Thiouridine (4SU), a uridine analog that is efficiently incorporated into RNA by all three cellular polymerases. After harvesting RNA, 4SU can be specifically biotinylated and newly made transcripts can be captured by streptavidin selection. The levels of nascent and newly made ORF59 and PAN RNA were unchanged between iSLK-WT and iSLK-ΔORF57 cells (Fig 3G). We conclude that the low expression of these viral RNAs in iSLK-ΔORF57 cells is primarily due to a post-transcriptional effect.

### PPD and ARS2-mediated decay target viral RNAs during lytic reactivation in iSLK-ΔORF57 cells

**Fig 4:**
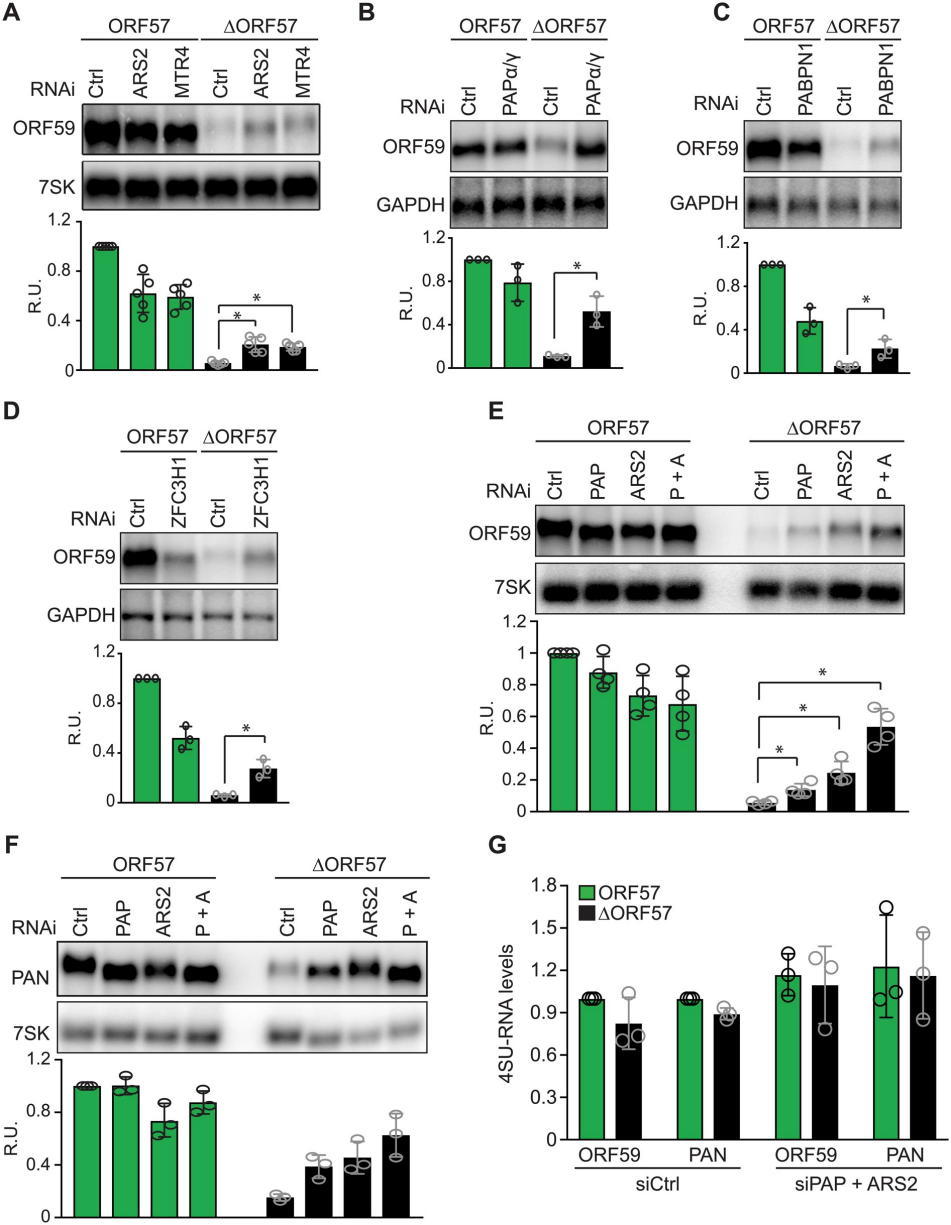
Inactivation of two RNA decay pathways restores viral RNA levels in iSLK-ΔORF57 cells. (A-E) Representative northern blots and quantification of ORF59 mRNA from iSLK-WT and iSLK-ΔORF57 transfected with a non-targeting control siRNA or a two-siRNA pool targeting ARS2 or hMTR4 (A), PAPα/γ (B), PABPN1 (C), ZFC3H1 (D), or ARS2/PAPα/γ co-depletion (E). Total RNA was purified 3 days after siRNA transfection, except for panel B where PAPα/γ were knocked down for 4 days. In all cases, cells were harvested 24 hours post lytic reactivation. All experiments included 3 biological replicates (n = 3) except for panels A and E, where quantifications were done from 5 and 4 replicates, respectively. 7SK RNA or GAPDH serves as loading control as indicated. (F) Representative northern blot and quantification (n = 3) of PAN RNA from iSLK-WT and iSLK-ΔORF57 transfected with a non-targeting control siRNA or a two-siRNA pool targeting PAPα/γ, ARS2 or both simultaneously. 7SK serves as loading control. (G) Quick 4SU pulse in iSLK-WT (green) and iSLK-ΔORF57 (black) cells transfected with a two-siRNA pool targeting both PAPα/γ and ARS2. In all graphs, mean values are plotted with standard deviation. Quantification was determined by first normalizing values to the corresponding loading control. iSLK-WT cells treated with control siRNAs was set as 1 and the rest of the values were calculated relative to it. Statistical analyses were two-tailed unpaired Student’s t-tests (*p<0.05).

Our pulse-chase reporter assays (Figs 1 and 2) are ideal for examining ORF57 effects on nuclear stability in a heterologous system. However, they are limited to a single nuclear RNA and are performed in the absence of other viral factors. Therefore, we next tested whether inactivation of various RNA QC factors can restore expression of ORF57-dependent genes in reactivated iSLK-ΔORF57 cells. We first validated protein, mRNA, and/or functional deletion of these factors in iSLK cells (S3A–S3G Fig). Upon ARS2 depletion, ORF59 mRNA levels were partially restored in iSLK-ΔORF57 cells (Fig 4A). We note that depletion of several of the factors tested, including ARS2, reduces ORF59 levels in iSLK-WT cells, presumably due to indirect effects of knocking down essential host genes on viral reactivation (Fig 4A). Upon siMTR4 treatment (S3A Fig), ORF59 mRNA levels were restored to levels similar to those observed upon ARS2 depletion (Fig 4A). In addition, we inactivated the NEXT complex by depleting RBM7 or ZCCHC8 in iSLK-WT and iSLK-ΔORF57 cells (S3C Fig). Twenty-four hours post lytic reactivation, levels of ORF59 as well as 2 other ORF57-dependent viral mRNAs, ORF8 and ORF9, were monitored by qRT-PCR. Although RBM7 and ZCCHC8 were efficiently depleted at the mRNA level (S3C Fig) and functionally inactivated as observed by increased expression of PROMPTs (S3F and S3G Fig), NEXT inactivation failed to restore the expression of ORF8, ORF9 or ORF59 in iSLK-ΔORF57 cells (S3H Fig). Consistent with our pulse-chase assays, these results suggest that ORF57 protects viral transcripts against an ARS2-dependent, NEXT-independent decay pathway during viral infection.

We next tested whether PPD promotes viral RNA decay during lytic reactivation. We knocked down PABPN1, PAPα/γ, or ZFC3H1 to inactivate PPD in iSLK-WT and iSLK-ΔORF57 cells (S3A–S3G Fig). Consistent with a role for PPD in viral RNA decay, depletion of PAPα/γ (Fig 4B), PABPN1 (Fig 4C) or ZFC3H1 (Fig 4D) partially restored ORF59 levels in iSLK-ΔORF57 cells following lytic reactivation. These data suggest that ORF59 is also targeted for degradation by PPD. Because the increases in ORF59 RNA upon PPD knockdown were only observed in the iSLK-ΔORF57 cells, these data support a role for ORF57 in protecting ORF59 from PPD during viral infection.

Our data suggest that viral transcripts are targeted for degradation by an ARS2-dependent decay pathway and PPD during lytic infection, but ORF57 counters these activities. To examine redundancy between the pathways, we inactivated both decay pathways by simultaneously knocking down PAPα/γ and ARS2 in iSLK-WT and iSLK-ΔORF57 cells. Following lytic reactivation, PAPα/γ and ARS2 co-depletion restored ORF59 mRNA levels to a greater extent than PAPα/γ or ARS2 alone in iSLK-ΔORF57 cells (Fig 4E). Similar results were observed with PAN RNA, another well characterized ORF57-dependent RNA (Fig 4F). These data suggest that inactivation of both decay pathways increases viral transcript levels by impairing their degradation.

The steady-state measurements of PAN RNA and ORF59 (Fig 4E and 4F) do not distinguish between changes in stability or transcription upon PPD and ARS2 co-depletion. We used a 10-min 4SU labelling assay to monitor new RNA synthesis in iSLK-WT and iSLK-ΔORF57 following PAPα/γ and ARS2 co-depletion. Simultaneous inactivation of both decay pathways did not affect the levels of newly made ORF59 or PAN RNA in iSLK-ΔORF57 or iSLK-WT cells compared to cells treated with control siRNA (Fig 4G). These data support the conclusion that ORF57 inhibits the actions of two host-mediated nuclear decay pathways, PPD and an ARS2-dependent decay pathway.

### ORF57 reduces hMTR4 recruitment to viral transcripts

**Fig 5:**
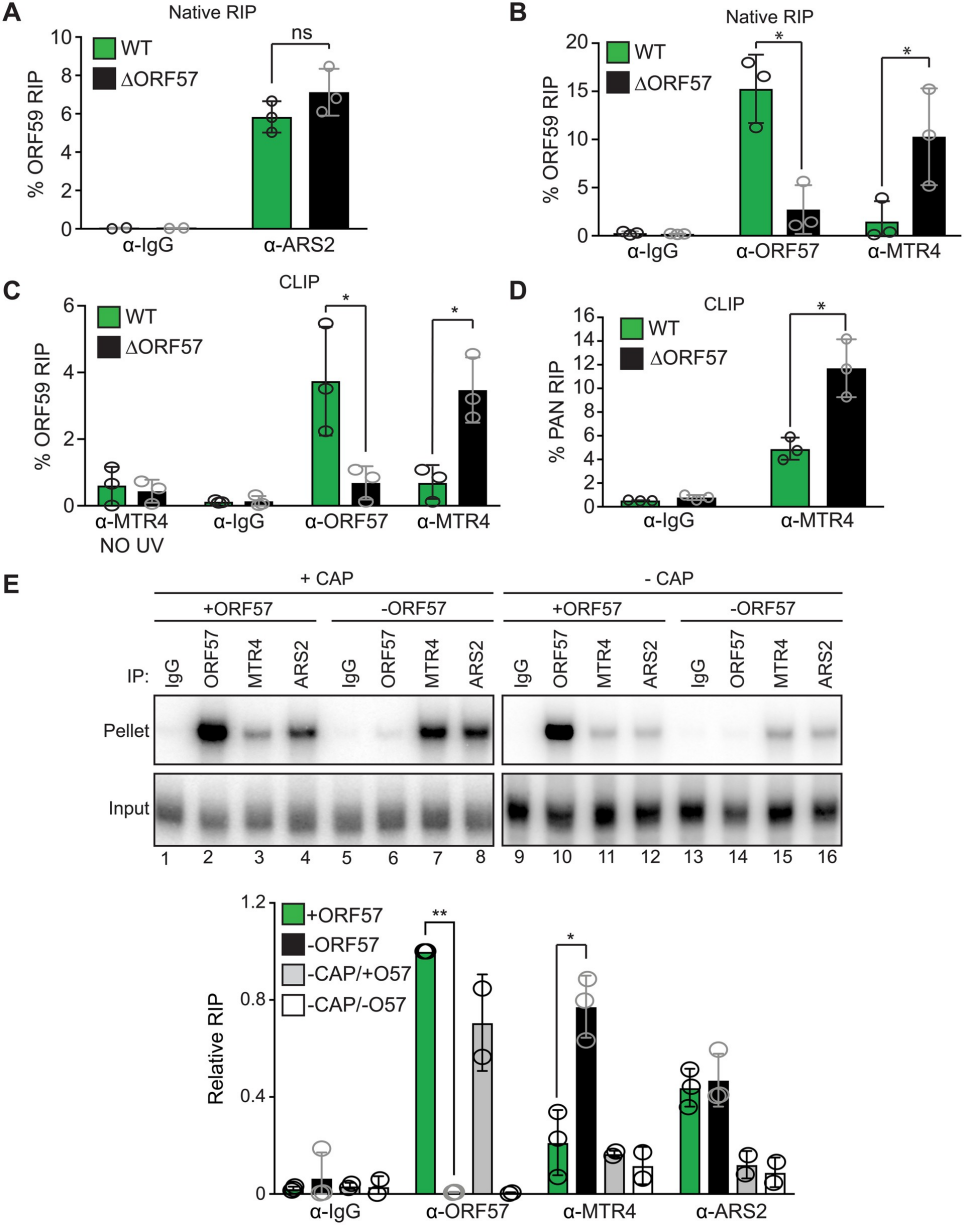
ORF57 reduces hMTR4 recruitment to viral transcripts. (A-B) Native RIP with IgG, ARS2, ORF57 or hMTR4 antibodies using extracts made from induced (24 hpi) iSLK-WT (green) or iSLK-ΔORF57 (black) cells. (C-D) CLIP with IgG, ORF57 or hMTR4 antibodies using extracts from induced (24 hpi) iSLK-WT (green) or iSLK-ΔORF57 (black) cells. The “no UV” and IgG samples show background of the assay. Values are average and error bars are standard deviations (n = 3). (E) Representative in vitro RNA IP assay. Extracts from cells expressing either an empty vector or ORF57 were incubated with an in vitro transcribed, radioactively labeled PAN RNA probe either capped or uncapped as indicated. Extracts were immunoprecipitated using IgG, ORF57, ARS2 or hMTR4 antibodies. 10% of input is shown. Bottom panel shows quantification of IPs using extracts expressing ORF57 (green), empty vector (black), an uncapped probe +ORF57 (gray) or an uncapped probe -ORF57 (white) (n = 3 for capped samples; n = 2 for uncapped). All values were calculated relative to anti-ORF57 in the ORF57 expressing extract. Statistical analyses were performed using two-tailed unpaired Student’s t-tests (*p<0.05, **p<0.01).

To elucidate the mechanism of ORF57 in RNA stabilization, we first focused our attention on ARS2. Previous reports showed that like the CBC-associated ARS2, ORF57 binds RNAs near their 5′ end [63] and interacts with the cap-binding protein 80 (CBP80) in an RNA-independent manner [46]. Therefore, we hypothesized that ORF57 protects viral transcripts by preventing ARS2 recruitment. To test this model, we performed native RNA immunoprecipitation (RIP). Lysates made from iSLK-WT and iSLK-ΔORF57 cells were immunoprecipitated using an anti-ARS2 antibody (S4A Fig) and ORF59 mRNA levels were monitored by qRT-PCR. Contrary to this hypothesis, ORF59 mRNA was similarly enriched in both iSLK-WT and iSLK-ΔORF57 lysates (Fig 5A), suggesting that ORF57 does not interfere with ARS2 recruitment to viral transcripts.

We next tested whether ORF57 affects hMTR4 recruitment to viral RNAs. Initially, we used native RIP to test the association of ORF57 (S4B Fig) and hMTR4 (S4C Fig) with ORF59 in iSLK-WT and iSLK-ΔORF57 lysates. As expected, ORF59 mRNA was efficiently co-immunoprecipitated from iSLK-WT lysates with anti-ORF57 antibodies (Fig 5B) [63]. However, ORF59 mRNA was more efficiently immunoprecipitated from iSLK-ΔORF57 lysates compared to iSLK-WT lysates with anti-hMTR4 antibodies (Fig 5B). These data suggest that hMTR4 recruitment is impeded in the presence of ORF57.

A major caveat of native RIP experiments is the reassortment of protein-RNA complexes that often occurs subsequent to cell lysis [75]. To circumvent this problem, we used a UV cross-linked RNA immunoprecipitation (CLIP) assay [76]. In this assay, live cells are exposed to UV light so observation of a UV-dependent crosslink demonstrates a protein-RNA interaction that occurs in cells. Prior lysis, iSLK-WT and iSLK-ΔORF57 cells were UV irradiated to crosslink proteins to RNA. After irradiation, cells were lysed and ORF57 (S4D Fig) or hMTR4 (S4E Fig) were immunoprecipitated. As observed in native RIP, ORF59 mRNA was significantly enriched in iSLK-ΔORF57 hMTR4 immunoprecipitates compared with the iSLK-WT (Fig 5C). Similarly, we observed significantly more PAN RNA co-immunoprecipitation with hMTR4 from iSLK-ΔORF57 lysates compared to the iSLK-WT (Fig 5D). These data further support the model that ORF57 inhibits hMTR4 recruitment to viral transcripts.

Finally, we used an *in vitro* approach to further test the model that ORF57 inhibits hMTR4 recruitment to RNA. Whole cell lysates from cells transfected with either an ORF57 expression plasmid or an empty vector control were incubated with an in vitro transcribed, radioactively labeled PAN RNA substrate. The probe included the ORF57 responsive element in the 5ʹ-end of PAN RNA and an m^7^G cap [60, 62]. Following incubation, lysates were immunoprecipitated using an IgG control, ORF57, hMTR4 or ARS2 antibodies and co-immunoprecipitated RNAs were detected and quantified by urea polyacrylamide gel electrophoresis (Fig 5E). As expected, the PAN RNA substrate efficiently co-immunoprecipitated with ORF57 (Fig 5E compare lanes 2 with 6 and see bar graph below). Consistent with our RIP results (Fig 5A), ARS2 immunoprecipitated PAN RNA similarly from lysate containing or lacking ORF57 (Fig 5E, compare lanes 4 with 8). Importantly, significantly more substrate was immunoprecipitated by anti-hMTR4 antibodies in lysates lacking ORF57 than those that contained ORF57 (Fig 5E, compare lanes 3 with 7). As expected from the ARS2 association with CBC, an uncapped substrate was not enriched after immunoprecipitation with ARS2 or hMTR4 (Fig 5E, compare lanes 11 with 15 and 12 with 16). As we previously observed, the ORF57 interaction is cap-independent (Fig 5E lanes 10 and 14) [62]. Taken together, these three independent assays strongly suggest that binding of ORF57 to viral transcripts protects them from host mediated RNA decay pathways by impeding hMTR4 recruitment.

### ALYREF overexpression restores PAN RNA and ORF59 expression during ΔORF57 lytic reactivation

**Fig 6:**
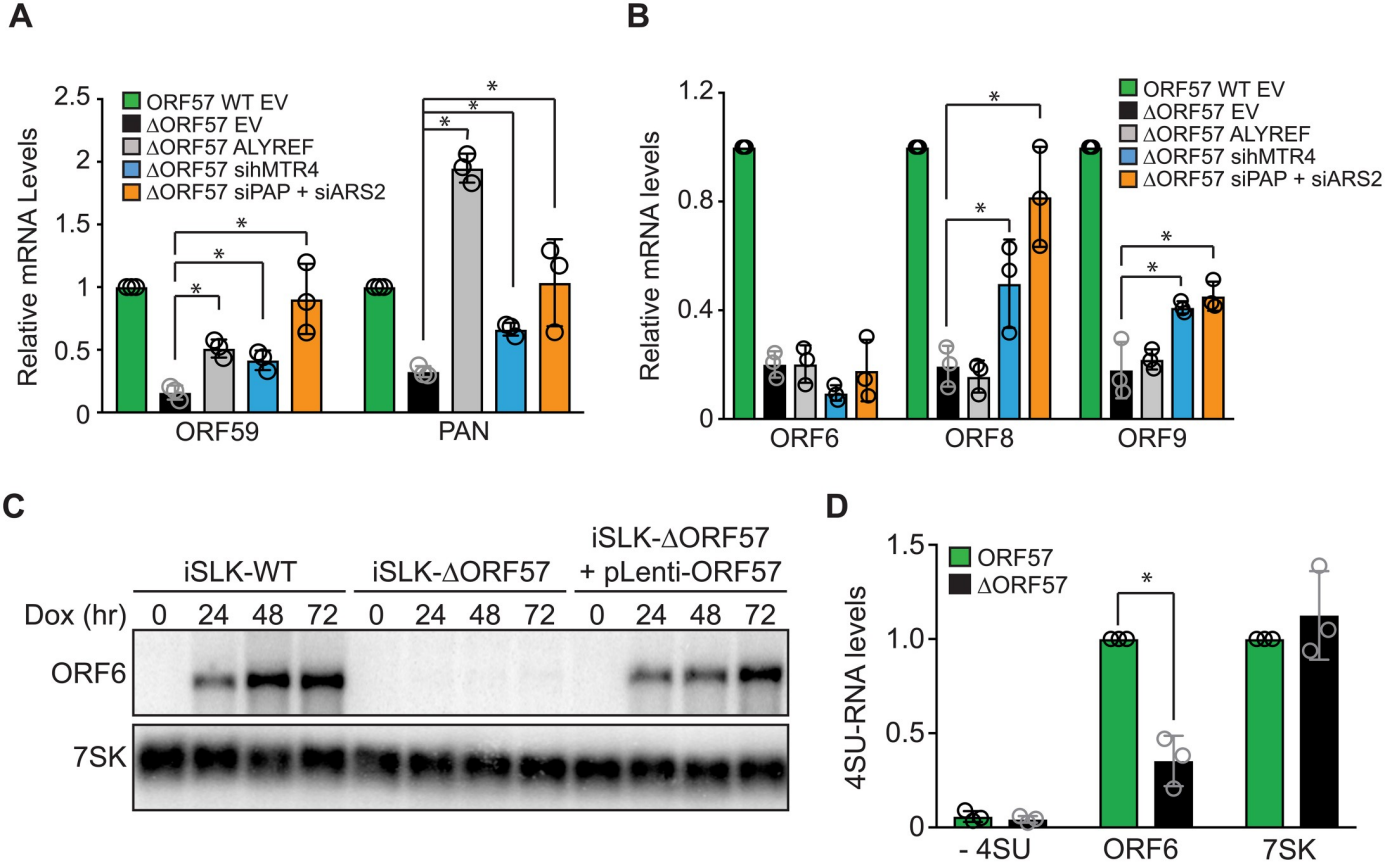
Gene-specific effects of ALYREF overexpression on ORF57-dependent viral RNAs. (A-B) Bar graphs showing results from qRT-PCR of ORF59 and PAN RNA (A), and ORF6, ORF8, and ORF9 (B) obtained from 293i-ORF57 (green) or 293i-ΔORF57 (black) cells. The cells were transfected with an empty vector (EV), or ALYREF overexpression construct (gray) as indicated. 293i-ΔORF57 cells were also subject to depletion of hMTR4 (blue), or ARS2/PAPα/γ co-depletion (orange). Lytic reactivation was achieved by transfecting cells with a plasmid expressing ORF50. Total RNA was harvested 36 hours post ORF50 transfection. Values are average and the error bars are standard deviations (n = 3). (C) Time course and representative northern blot of ORF6 from iSLK-WT, iSLK-ΔORF57 and iSLK-ΔORF57 cells transduced with a lentivirus expressing ORF57. 7SK serves as loading control. (D) Ten-minute 4SU pulse labeling in iSLK-WT (green) and iSLK-ΔORF57 (black) cells. ORF6 mRNA levels were measured by qRT-PCR. Quantification was performed as in Fig 3G. The “- 4SU” values were calculated relative to ORF6 WT. Values are average and error bars are standard deviations (n = 3).

We previously reported that ORF57-dependent stabilization of PAN RNA depends on the export adaptor ALYREF [64]. In fact, tethering ALYREF to the 5′ end of PAN RNA is sufficient to increase PANΔENE stability in the absence of ORF57 [64]. More recently, Fan et al. reported that ALYREF competes with hMTR4 for association with ARS2 and the outcome of this competition determines the fate of the transcript [77]. That is, if ALYREF binds ARS2 the transcript is stable, but if hMTR4 outcompetes ALYREF the RNA is degraded. These observations suggest that ORF57 recruits ALYREF to viral transcripts to prevent the association of ARS2 with hMTR4. To test this idea, we overexpressed ALYREF, depleted hMTR4, or co-depleted PAPα/γ and ARS2 in 293i-ΔORF57 cells (S5 Fig) and monitored the expression levels of ORF59 and PAN RNA by qRT-PCR 36 hours after ORF50 co-transfection to promote lytic reactivation. 293i-WT and 293iΔORF57 cells were generated by infecting HEK293 cells with virus made from iSLK-WT or iSLK-ΔORF57 cells complemented in *trans* with an ORF57 expression plasmid. We used 293i cells because they allow robust transfection and overexpression compared to iSLK derivatives. Interestingly, ALYREF overexpression partially restored ORF59 levels in 293iΔORF57 while PAN RNA accumulated to levels even higher than those observed in 293i-WT cells (Fig 6A grey bars). As expected, hMTR4 depletion and PAPα/γ/ARS2 co-depletion also led to increases in ORF59 and PAN RNA in 293i cells (Fig 6A, blue and orange bars). These data support a role for ALYREF in regulating the stability of viral transcripts.

### ORF57 deletion affects viral gene expression by multiple mechanisms

ORF57 is essential for the expression of a number of viral transcripts, but it remains unknown whether ORF57 affects the stability of all of these transcripts or changes their expression by a distinct direct or indirect mechanism. Verma et al. identified ORF6, ORF8 and ORF9, as ORF57 targets [69], so we monitored their expression levels upon hMTR4 knockdown, PAPα/γ and ARS2 co-depletion or ALYREF overexpression. hMTR4 depletion and PAPα/γ/ARS2 co-depletion increased the expression of ORF8 and ORF9 in 293i-ΔORF57 cells (Fig 6B, blue and orange bars), supporting a role for ORF57 in their nuclear stability. However, none of the transcripts increased upon ALYREF overexpression suggesting that ORF57 inhibits their decay by an ALYREF-independent mechanism (Fig 6B, gray bars) (see Discussion).

ORF6 expression was not increased in 293i-ΔORF57 cells by hMTR4 depletion, PAPα/γ/ARS2 co-depletion, nor by ALYREF overexpression. In iSLK-ΔORF57 cells, ORF6 mRNA levels are undetectable by northern blot, but they can be restored by transducing cells with lentivirus expressing ORF57 (Fig 6C). These data confirm that ORF6 expression is ORF57 dependent and they demonstrate that our ΔORF57 bacmid does not have a spontaneous mutation that prevents ORF6 expression. Next, we determined whether ORF6 transcription rate is compromised in iSLK-ΔORF57 cells. A 10-minute 4SU labelling assay showed that 4SU incorporation into ORF6 is significantly reduced in iSLK-ΔORF57 cells (Fig 6D). We conclude that ORF6 transcription is compromised by ORF57 deletion, but whether this is due to direct effects of ORF57 on transcription or indirect effects of ORF57 deletion on the progression through the lytic gene expression cascade remains unknown.

## Discussion

**Fig 7:**
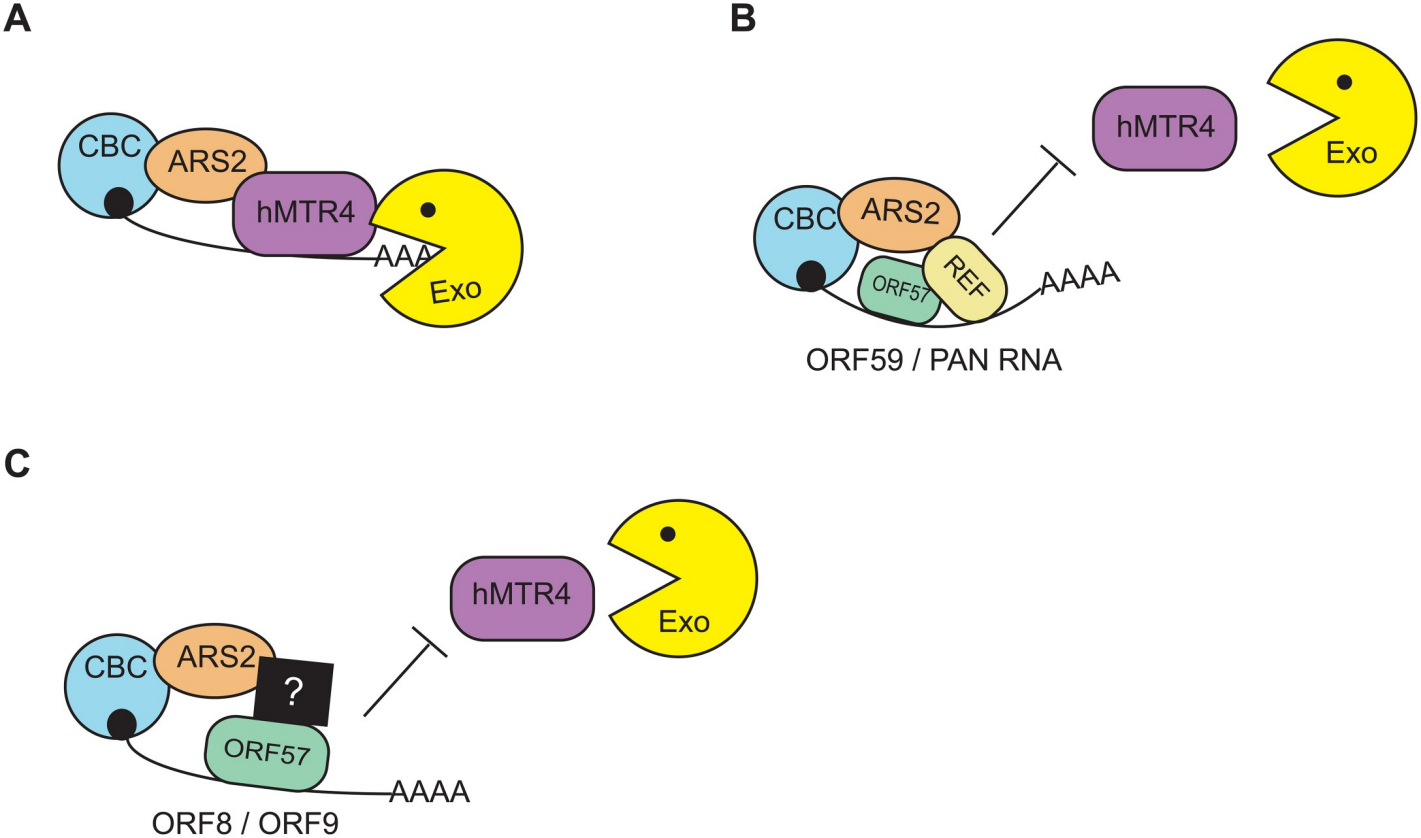
Model of ORF57-mediated protection of KSHV transcripts. (A) In the absence of ORF57, hMTR4 interacts with ARS2 and recruits the nuclear exosome to viral transcripts to degrade them. (B) When ORF57 is expressed, it directly binds to viral transcripts and recruits the export adaptor ALYREF. The ORF57 recruitment of ALYREF allows it to outcompete hMTR4 for ARS2 binding to protect viral transcripts from exosome-mediated degradation. (C) For ALYREF-independent, ORF57-dependent RNAs (e.g. ORF8 and ORF9), we speculate that ORF57 recruits an unknown cellular factor(s) (black square) which prevents hMTR4 recruitment to viral RNAs. See text for more details. For simplicity, PPD factors were omitted from the diagram.

The KSHV ORF57 protein functions to enhance the nuclear stability of intronless viral transcripts by protecting them from cellular RNA QC pathways. In this article, we report three findings regarding ORF57 mechanisms in RNA QC. First, ORF57 modulates the susceptibility of viral transcripts to two host-mediated nuclear RNA decay pathways, PPD and an ARS2-dependent decay pathway. Second, the presence of ORF57 reduces the recruitment of the nuclear exosome co-factor hMTR4 to viral transcripts. Third, we implicate the ORF57 co-factor ALYREF in the inhibition of nuclear decay for a subset of viral transcripts. Based on these data, we propose the model in Fig 7. In the absence of ORF57, ARS2 efficiently recruits hMTR4 to ORF59 and PAN RNA to promote exosome-mediated decay (Fig 7A). When ORF57 is present, it binds to ORF59 and PAN RNA and recruits ALYREF to the transcripts. Consequently, ALYREF interacts with ARS2, prevents hMTR4 recruitment, and protects the viral transcripts from degradation (Fig 7B). Consistent with this model, a competitive interaction between ALYREF and hMTR4 for ARS2 binding regulates the fate of a subset of HeLa transcripts. Interaction between ALYREF and ARS2 results in increased stability and export of the RNA, while an hMTR4-ARS2 interaction promotes exosome-mediated degradation of the transcript [77]. In a similar fashion, we propose ORF57 promotes viral transcript stability by shifting the competitive balance in favor of ALYREF over hMTR4.

At least two ORF57-dependent transcripts, ORF8 and ORF9, undergo PPD and ARS2-mediated decay, but their expression cannot be restored upon ALYREF over-expression in ORF57 knockout cells (Fig 6B). One interpretation of these data is that ORF57 recruits an unidentified factor(s) that allows these transcripts to avert hMTR4 recruitment (Fig 7C). An interesting candidate is SRSF3, an splicing factor that is involved in regulation of splicing and mRNA nuclear export. Like ALYREF, SRSF3 promotes the recruitment of the Nuclear Export Factor 1 (NXF1) to nuclear mRNAs to couple RNA processing to nuclear export [78–80]. Moreover, SRSF3 interacts with ORF57 to modulate splicing of KSHV’s K8β RNA [81]. Interestingly, SRSF3 has been reported to modulate the degradation of intronless transcripts by interaction with EB2, the ORF57 homolog in Epstein Bar virus [82]. However, the role of SRSF3 as an ORF57 co-factor needed for viral transcript stabilization has yet to be examined. Therefore, it is possible that ORF57 recruits SRSF3 to viral transcripts to promote their stability through the RNA export machinery. More broadly, this model suggests that ORF57 uses distinct co-factors to increase the stability of specific subsets of viral RNAs in the nucleus. Defining these pathways will reveal new proteins important for viral gene expression and define new roles for host proteins in nuclear RNA stability.

Our model builds on several previous observations that ORF57 recruits ALYREF to increase viral transcript stability independent of its role in nuclear export. Our lab showed that PAN RNA nuclear abundance is increased upon recruitment of ALYREF in an ORF57-dependent manner [64]. Importantly, neither recruitment of ALYREF by ORF57 nor direct tethering of ALYREF to PAN RNA promotes nuclear export of PAN RNA. Similarly, Li et al. showed that complementing an ORF57-null KSHV bacmid with a plasmid expressing an ALYREF-binding deficient ORF57 mutant (PP208/211AA) results in reduced levels of PAN RNA as compared to complementation with wild-type ORF57 [83]. Taken together, these data strongly suggest that ALYREF can promote PAN RNA stability in the absence of export.

PAN RNA is a nuclear, non-coding RNA that does not undergo nuclear export. Therefore, it is possible that for viral transcripts that are readily exported (e.g. ORF59), ALYREF binding promotes their nuclear export, reducing their exposure to nuclear decay pathways and increasing the abundance of the transcript. However, our data here as well as previously published reports suggest this is not the case. First, in the absence of ORF57, ORF59 is subject to similar nuclear decay pathways as PAN RNA (Fig 4), and it responds similarly to ALYREF overexpression (Fig 6). Second, several groups showed that ORF57 increases both nuclear and cytoplasmic mRNA levels for ORF59 or other mRNA reporters [56, 66, 68, 83–85]. Interestingly, complementation experiments showed that ALYREF-binding deficient ORF57 maintained the ability to increase ORF59 levels, but to a lesser extent than with the wild-type [83]. The lack of a complete abrogation could be due to residual ALYREF binding, or to compensation by other ORF57 co-factors that promote ORF59 stability in the absence of ALYREF (Fig 7C). These observations support our proposed model that ORF57 recruits ALYREF to protect ORF59 and PAN RNA from hMTR4 (Fig 7B).

Unlike other ORF57-dependent transcripts, ORF6 expression cannot be restored in ORF57-null cells upon inhibition of nuclear decay. Instead, our data suggest that ORF6 is regulated transcriptionally (Fig 6D). Because ORF57 is a multifunctional protein that has been implicated in transcription regulation [50, 51], it may directly promote ORF6 transcription. Alternatively, ORF57 could regulate ORF6 expression indirectly. That is, ORF57 promotes the expression of another KSHV gene(s) that is essential for ORF6 transcription. While further experimentation is needed to distinguish between these models, these observations underscore that ORF57 uses multiple mechanisms to regulate viral gene expression.

The recent definition of the NEXT, CBCA, and PAXT complexes represents a major step forward in understanding nuclear RNA decay in mammalian cells [17, 19, 20, 24, 27]. Despite this progress, the mechanisms used by these factors to promote the decay of transcripts is unclear. In particular, the overlap or specificity among both the protein components of each complex and their RNA targets remain ambiguous. Our data show that depletion of the NEXT components ZCCHC8 or RBM7 do not increase the stability of PANΔENE in pulse chase assays (Fig 2A and 2B and S2A and S2B Fig) nor did it restore the expression of viral transcripts generated from an ORF57-null bacmid (S3H Fig). Thus, we conclude that NEXT is not a key contributor to the surveillance of KSHV RNAs.

During viral infection, the deletion of ORF57 results in viral RNAs being subject to two decay pathways, PPD and another involving ARS2 (Fig 4). PPD targets poorly exported, polyadenylated transcripts including ncSNHGs, pri-miRNAs, lncRNAs and a subset of PROMPTs [18, 21]. As part of CBC-NEXT complex, ARS2 targets short lived nuclear transcripts including PROMPTs and enhancer RNAs [17, 19]. As KSHV transcripts share features of transcripts targeted by both pathways, it is reasonable that infected cells utilize both pathways to eliminate foreign viral RNAs. Interestingly, in contrast to the reactivation studies, our reporter assays suggest that ORF57 does not directly inhibit PPD, but instead preferentially inhibits ARS2-mediated decay (Figs 1 and 2). We can think of two non-mutually exclusive explanations for these apparently contrasting observations. First, ORF57 may protect from PPD in the reporter assays, but our interpretations are confounded by incomplete knockdown of PPD and the effects of ORF57 on ARS2-mediated decay. Second, the reporter assays are performed in the absence of other viral factors and are limited to one specific RNA. As such, ORF57 may preferentially protect PANΔENE from ARS2-mediated decay, but it plays a broader role in RNA stabilization in the context of viral infection. This broader role could be a direct effect dictated by features like transcript length or specific sequences. Alternatively, the role could be an indirect effect of ORF57 loss on the production of other viral proteins or on host responses. Further work is required to distinguish these hypotheses.

The interrelationships between PPD and ARS2-mediated decay processes for viral or host RNAs remain somewhat obscure. We further note that the abbreviations PAXT and PPD were proposed independently [18, 19], but they have been interpreted by us and others to describe the same pathway; PAXT refers to the protein complex (PABPN1, ZFC3H1, and hMTR4) while PPD to the decay process. Importantly, ARS2 has been reported to be involved in PAXT recruitment to unstable transcripts [19]. Therefore, if ARS2 is required for PAXT function, and if PAXT and PPD describe the same pathway, then depletion of ARS2 should be sufficient to eliminate both pathways. However, when we simultaneously deplete cells of PAPα/γ and ARS2, viral transcripts are stabilized to a greater extent than depletion of either PAPα/γ or ARS2 alone (Figs 2E & 4E). Admittedly, these observations could stem from incomplete knockdown being synergistic between two factors in the same pathway. Nonetheless, we interpret these data to imply the existence of two independent pathways, but the distinction between the two pathways is unclear. In one possibility, PPD is separable from PAXT. Consistent with this, PAPα/γ have yet to be directly linked to PAXT, but the PAPs are a defining component of PPD [18]. Alternatively, PPD and PAXT may represent the same process, but ARS2 is not be absolutely required for PAXT activity. Considerable efforts will be necessary to define these pathways, but the data presented here support the model that ORF57 protects viral transcripts from processes involving ARS2, PABPN1, hMTR4, ZCFC3H1 and PAPα/γ (Fig 4) that otherwise would target viral RNAs for degradation.

Our data suggest that ORF57 neutralizes the action of nuclear RNA decay pathways by preventing hMTR4 interaction with viral transcripts (Fig 4). Interestingly, this is not the first report indicating an antiviral role for hMTR4 and the RNA exosome. In response to cytoplasmic RNA virus infection, hMTR4 and hZCCHC7 translocate from the nucleus to the cytoplasm where they aid in exosome-mediated degradation of viral RNAs [86, 87]. Similarly, we show that depletion of hMTR4 restores the expression of KSHV transcripts generated from an ORF57-null bacmid (Figs 4A, 6A and 6B). These data suggest that hMTR4 may be a cell intrinsic factor that represses viral gene expression during infection. As such, it is not surprising that KSHV ORF57 evolved to overcome hMTR4-dependent cellular RNA decay pathways.

It remains to be determined how cellular RNA decay pathways affect the expression of most KSHV genes. As KSHV genes are transcribed and processed in the host nucleus, all of the resulting transcripts must avoid RNA QC pathways. A complete understanding of these mechanisms will be challenging as they involve RNA decay, processing, and its coordination with transcription. In fact, the exosome and its cofactors have been implicated in regulating transcription of cellular and viral genes [88, 89]. Therefore, it will be interesting to determine how ORF57-mediated regulation of RNA stability interfaces with transcription and processing globally.

## Materials and methods

### Cell culture

293A-TOA [59], 293i and iSLK cells (gift from Dr. Rolf Renne, University of Florida) were grown at 37°C with 5% CO_2_ in DMEM (Sigma) supplemented with 10% Tet-Free fetal bovine serum (FBS, Atlanta Biologicals), 1x penicillin-streptomycin (Sigma), and 2 mM L-glutamine (Fisher). iSLK-WT and iSLK ΔORF57 cells were grown in the presence of 0.1 mg/mL G418 (Fisher), 1 μg/mL puromycin (Sigma) and 50 μg/mL hygromycin. 293i cells were maintained in media containing 50 μg/mL hygromycin. HEK293 cells (gift of Dr. Joan Steitz, Yale University) were cultured in the same conditions, but regular FBS (Sigma) was used instead.

### Generation of ORF57-null bacmid iSLK-ΔORF57 cells

The markerless Red Recombination system was used to make the BAC16-ΔORF57 bacmid [90–92]. Amplification and manipulation of bacmids were performed in GS1783, a recA^-^ recombination-deficient *E*. *coli* strain that contains a heat-inducible Red recombinase system and an arabinose-inducible I-SceI homing endonuclease gene. Bacteria were grown at 30–32° except where noted. GS1783 bacterial cells and a plasmid containing a kanamycin-resistance gene with an adjacent I-SceI site (GS1439) were generous gifts of Dr. Greg Smith (Northwestern University) and Dr. Nikolaus Osterrieder (Freie Universität Berlin).

For the first step of the two-step Red recombination system, we performed PCR using primers NC1175 and NC1176 with GS1439 as a template to create a PCR product that has an I-SceI site next to the kanamycin resistance gene. In addition, the primers contain unique left and right homology regions to target the ORF57 locus of BAC16. They also contain the desired ORF57 deletion sequence flanking the I-SceI containing kanamycin resistance gene for recombination in step 2 (see below). We obtained GS1783 cells carrying BAC16 from Dr. Jae Jung (University of Southern California) [72]. These bacteria (3mL) were grown to an OD_600_~0.5 in 30 μg/mL chloramphenicol LB broth, then transferred to 42° water bath for 5 min to induce recombination enzymes and subsequently chilled in an ice bath for 20 min. The cells were harvested, washed twice in ice-cold 10% glycerol, then resuspended in 50 μL of the same. One hundred nanograms of the gel-purified I-SceI/kanamycin/ORF57 targeting PCR product was then electroporated in 1 mm cuvettes (1.5 kV, 25 μF, and 200 Ω). The bacteria were then grown in 1 mL of LB for 2hr/30° and plated on LB plates with chloramphenicol and kanamycin. Colonies were allowed to grow for two days then individual clones were selected and bacmid DNA was isolated by miniprep using standard techniques.

The clones were screened by three independent validation strategies. Proper targeting was verified by colony PCR using primers NC1179/NC1178 and NC1177/NC497 (S4 Table) which span the left and right junctions of the targeting locus. DNA from positive clones was then digested with NheI and analyzed by pulsed-field gel electrophoresis (PFGE) as described [89]. This step is required for the selection of only those clones that retain full-length terminal repeats (TR), which are essential for replication. Due to their repetitive nature, the TRs are often lost during the recombination step.

After selection of clones with proper targeting and full-length TRs, we performed the second step of the Red recombination approach that removes the kanamycin resistance gene and creates the ORF57 deletion. Overnight cultures of the positive clones were diluted (10 μL into 1 mL of LB+chloramphenicol), grown for 2 hr at 30°, then diluted 1:1 with LB/chloro containing 2% arabinose to induce I-SceI expression. This generates a dsDNA break at the I-SceI site flanking the kanamycin-resistance gene. One hour after arabinose addition, the cells were shifted to 42° for 5 min then placed at 30° for an additional 3 hours. This step induces the recombination machinery to drive homologous repair between the duplicated ORF57-deletion sequences that flank both sides of the kanamycin-resistance gene. Colonies were grown on chloramphenicol plates lacking kanamycin and screened by: 1) verification of loss of kanamycin-resistance, 2) PCR using NC1179 and NC497, 3) PFGE. Bacmid DNA was prepared from the positive clones using the Qiagen Large-Construct kit and the resulting bacmids were once again validated with PFGE and the junction was sequenced to verify the precise 33-bp ORF57 deletion. BAC16 and deletion bacmid were then transfected into iSLK cells using FuGene and selected with 300 μg/mL of hygromycin [72]. Multiple independent transfections and bacmid deletion clones were tested and the H6 iSLK-ΔORF57 were used for subsequent experiments because they carried similar KSHV DNA content to the wild-type BAC16 and were complemented by ORF57 (Fig 3).

### siRNA and DNA transfections

All the proteins studied here are essential, so care was taken to optimize siRNA conditions to produce the most efficient functional knockdowns with the lowest cell toxicity. iSLK cells were transfected with 20 or 40 nM siRNA (Silencer Select, Ambion) using RNAiMAX transfection reagent (Invitrogen) per manufacturer’s instruction. Specifically, we used final concentrations of 40 nM siRNAs for ZFC3H1, RBM7, ZCCHC8 and hMTR4 and 20 nM siRNAs for PABPN1 and ARS2. For PAPα/γ, we used 20 nM each of siRNAs that target PAPα or PAPγ for a total of 40 nM siRNA. Twenty-four hours post siRNA transfection, cells were split into new plates and allow to grow for another 24 hours, after which doxycycline and NaB was added to induce lytic reactivation. Total RNA was harvested 72 hours post siRNA transfection and 24 hours post lytic reactivation. For PAPα/γ knockdown, we observe a more robust effect after 4 days of silencing (Fig 4B). However, for PAPα/γ/ARS2 co-depletion, we performed knockdowns for 3 days as ARS2.

For pulse-chase assays in 293A-TOA cells, knockdowns were performed following the conditions described for iSLK cells. Twenty-four hours post transfection, cells were split into new plates and allow to grow for another 24 hours, after which they were transfected with the appropriate reporter construct. The one exception was hMTR4. In this case, cells were allowed to grow for 48 hours before being transfected with the reporter construct. For DNA (PANΔENE reporter) transfections, cells grown in a single well of a 12-well plate were transfected with 0.8 μg of DNA using TransIT-293 (Mirus) following manufacturer’s protocol. siRNAs are listed in S1 Table.

### Transcription pulse-chase assays

Transcription pulse-chase assays were performed as previously described [22, 61]. Briefly, 24 hours post siRNA transfection, 293A-TOA cells were seeded in 12-well plates. Twenty-four hours later, cells were transfected with 200 ng of a reporter construct expressing TetRP-driven PANΔENE and 600 ng of Fl-ORF57 or pcDNA3. Cells were grown in the presence of 5 ng/mL of doxycycline (dox) to repress transcription. Twenty-four hours after DNA transfection, cells were washed twice with phosphate-buffered saline (PBS) with calcium/magnesium (Sigma) and incubated in dox-free media for 2 hours. Transcription was repressed by adding dox (50 ng/mL final concentration). Total RNA was harvested at specified times using TRI reagent (Molecular Research Center), and analyzed by northern blot.

### Northern blotting

RNA was resolved on 1.2% formaldehyde–agarose gels using standard procedures [93]. After transfer to nylon membranes (Hybond N+, GE Healthcare) blots were probed in Church’s hybridization buffer overnight at 65°C. Probes were transcribed in vitro. A typical probe reaction consisted of 40 mM Tris pH 7.5, 6 mM MgCl_2_, 4 mM spermidine, 10 mM DTT, 200 ng T7-driven template, 0.5 mM ATP, CTP, and GTP, 2 U/μL RNasin, 50 μM UTTPαS (TriLink Biotechnologies), 25 μCi of α-^32^P-UTP (800 Ci/mmol), and T7 RNA polymerase. Probes were made from PCR-generated DNA templates or by enzymatic digestion of a plasmid (S2 Table). Bands were detected using a Typhoon FLA 9500 Phosphorimager (GE Healthcare) and quantified using ImageQuant v5.2.

### Immunobloting

Cells were lysed in buffer containing 100 mM NaCl, 50 mM Tris-HCl pH 7.4, 1% Triton X-100, 1X Protease Inhibitor cocktail (PIC) (Calbiochem) and 250 μM PMSF. Proteins were resolved by SDS-PAGE and western blotted using standard procedures. Antibodies used are given in S3 Table. Quantitative westerns were performed using infrared detection with an Odyssey Fc and quantification was performed using ImageStudio software (LI-COR Biosciences). For ORF57 immunoprecipitations, specific bands were detected using Clean-Blot detection reagent (Thermo).

### Quantitative RT-PCR

RNA was harvested using TRI reagent according to the manufacturer’s protocol. Following extraction, RNA was treated with RQ1 DNase (Promega). Random hexamers were used to prime cDNA synthesis with MuLV reverse transcriptase (New England Biolabs). Real-time reactions used iTaq Universal SYBR Green Supermix (Biorad). Primers are listed in S4 Table.

### KSHV reactivation and infection

Lytic reactivation of iSLK derived cells was achieved by adding doxycycline (1 μg/ml) and NaB (1mM). Ninety-six hours post induction, media from iSLK-WT, iSLK-ΔORF57 and iSLK-ΔORF57 + pLenti-ORF57 cells were collected, centrifuged for 5 min at 1000 xg and passed through a 0.45 um filter. Polybrene was added (8 μg/mL final concentration) and 300 μL of pre-warmed virus-containing media were applied to HEK293 cells grown in a 12-well plate. Cells were centrifuged for 45 min at 30°C and then incubated in 5% CO2 at 37°C for 2 hours. After this, media was replaced and 24 hours later, cells were analyzed by FACS.

### Native RIP

Native RIP was performed as previously described [94]. One 15-cm plate was used per condition. Twenty-four hours post lytic induction, iSLK-WT and iSLK-ΔORF57 cells were harvested by trypsin, quenched with media and washed with PBS. Cell pellets were resuspended in 400 μL of RSB100-T (10 mM Tris-HCl pH 7.5, 100 mM NaCl, 2.5 mM MgCl_2_, 0.5% Triton X-100) supplemented with protease inhibitors and RNasIn Plus (Promega). Next, CaCl_2_ was added to 5 mM with 45U of RQ1 DNase and incubated for 15 min at 25°C. For RNA digestion, Micrococcal Nuclease (2x10^6^ Gel Units/mL; NEB) was diluted 1:200 and 10 μL of this freshly diluted stock was added to the extract. RNA digestion proceeded at 25°C for precisely 10 min, after which 50 μL of 300 mM EGTA was added to stop the reaction. After clarification of the lysate by centrifugation at 16000 xg for 10 min, the protein-RNA complexes were immunoprecipitated for 1 hr with anti-hMTR4, ARS2, ORF57, ALYREF or IgG1 antibody (S3 Table). Twenty microliters of washed Protein A Dynabeads were added to each sample for 1 hr. Beads were then washed with ice-cold RSB100-T five times and then eluted with a proteinase K solution (0.1 mg/mL Proteinase K, 0.1% SDS, 20 mM Tris-HCl pH 7.5, 5 mM EDTA, 0.1 mg/mL competitor RNA) for 30 min at 37°C. Following digestion, samples underwent phenol:chloroform:isoamyl alcohol (25:24:1) (PCA) extraction and were ethanol precipitated. The resulting RNA was then used for quantitative RT-PCR.

### CLIP

CLIP was performed as previously described [63, 76] with modifications. Twenty-four hours post lytic reactivation, iSLK-WT and iSLKΔORF57 cells grown in 15 cm plates were washed 2X with PBS. After the last wash, 5 mL of PBS were added, and cells were irradiated at 400 mJ/cm^2^ on a Spectrolinker^tm^ XL-1500 UV cross-linker. After crosslinking, cells were harvested by trypsin, quenched with media and washed with PBS. Cells were spun down at 700 xg for 3 min and pellets were resuspended in 100 μL of SDS lysis buffer (50 mM Tris pH 6.8, 1 mM EDTA, 0.5% SDS, 0.125 mg/mL Heparin, 1 mM DTT, 1mM PMSF and 1X protein inhibitor). Samples were heated at 65°C for 5 min and immediately placed on ice for 2 min. After this, 400 μL of RIPA correction buffer (62.5 mM Tris pH 8.0, 1.25% Igepal, 0.625% sodium deoxycholate, 2.25 mM EDTA, 187.5 mM NaCl, 0.125 mg/mL heparin, 1 mM DTT, 1 mM PMSF, 1X protease inhibitor) were added to each sample and run through a QIAGEN QiaShredder column 2 times. Next, CaCl_2_ was added to 5 mM with 45U of RQ1 DNase and incubated for 15 min at 25°C. For RNA digestion, Micrococcal Nuclease (2x10^6^ Gel Units/ml) was diluted 1:200 and 10 μL of this freshly diluted stock was added to the extract. RNA digestion proceeded at 25°C for precisely 10 min, after which 34 μL of 300mM EGTA was added to stop the reaction. After clarification of the lysate by centrifugation at 16000 x g for 10 min, the Protein-RNA complexes were immunoprecipitated for 1 hr with anti-hMTR4, ORF57 or IgG1 antibody. Twenty microliters of washed Protein A Dynabeads were added to each sample for 1 hr. Beads were then washed with RIPA buffer five times and then eluted as described for native RIP. Following elution, samples underwent PCA and chloroform extraction and were ethanol precipitated. The resulting RNA was then analyzed by qRT-PCR.

### Quick-pulse transcription assays

Seventy-two hours after PAPα/γ and ARS2 knockdown and 24 hours post lytic induction, iSLK-WT and iSLK ΔORF57 cells were treated with 500 μM 4-Thiouridine (4SU) for 10 minutes. RNA was then harvested using TRI reagent and treated with RQ1 DNase. 80 μg of RNA were biotinylated at room temperature for 3 hours in a solution of 10 mM Tris-HCl pH 7.5, 1mM EDTA, 0.1% SDS and 0.2 mg/mL Biotin-HPDP (Thermo Fisher). Biotinylated RNA was extracted with chloroform three times, and then ethanol precipitated. Streptavidin selection was performed using 20 μL of Dynal MyOne Streptavidin T1 bead slurry (Thermo Fisher). Prior to incubation with sample, beads were washed 3 times with MPG 1:10-I (100 mM NaCl, 1 mM EDTA, 10 mM Tris-HCl pH 7.5, 0.1% Igepal) and then blocked for 1 hour in the same solution supplemented with 0.1 μg/μL poly(A), 0.1 μg/μL cRNA, 0.1 μg/μL ssDNA and 0.1% SDS. Biotinylated RNA and blocked beads were nutated for 1 hour at room temperature and then washed 10 times as previously described [95]. Samples were eluted twice with 0.1 M DTT in MPG 1:10-I for 15 minutes. Elutions were combined and extracted, first, with PCA and then two more times with chloroform. RNA was ethanol precipitated and analyzed by qRT-PCR.

### In vitro RNA immunoprecipitation

Body labeled RNA probe was made using a plasmid expressing KSHV PAN RNA as template in a 20 μL reaction containing 50 units of T7 RNA polymerase, 2.5 μL of ^32^P-UTP (800 Ci/mmol), 1U/μL of RNasIn Plus, 1 mM each of ATP, CTP and GTP, 50 μM UTP and transcription buffer (40mM Tris pH 7.5, 6 mM MgCl_2_, 4 mM spermidine and 10 mM DTT). Reaction was incubated at 37°C for 2 hours and then run through a MicroSpin G-50 column (GE). RNA was ethanol precipitated, resuspended in 15 μL of water and capped using the Vaccinia Capping system (NEB) following manufacturer’s recommendations. Capped RNA was extracted 1X with PCA, 1X with chloroform, ethanol precipitated and, then, separated on a urea-PAGE. RNA band of the correct size was excised and eluted with G-50 buffer (20 mM Tris pH 6.8, 0.3 M NaOAc, 2 mM EDTA and 0.025% SDS) overnight. Eluted RNA was extracted with PCA, ethanol precipitated and resuspended in water. RNA yield was determined by scintillation counting.

Whole cell extracts were made from HEK293 cells transfected with either pcDNA3 or pcFL-ORF57II as previously described [62]. The binding reactions were performed at 30°C for 30 min and consisted of 21 μL of whole cell extract, 2.5 μL of 10X binding buffer (200 mM Hepes pH 7.9, 1.5M KCl, 1% Triton X-100, 5 mM MgCl_2_, 50 mM DTT, 10 mM ATP and 3 mg/mL torula yeast RNA), 0.5 μL RNasin Plus and 10^5^ CPM of radioactively labeled RNA probe. Once the binding reactions were completed, volumes were increased to 250 μL by adding 225 μL of 1X IP buffer (20 mM Hepes pH 7.9, 150 mM KCl, 0.1% Triton X-100, 0.5 mM MgCl_2_, 2.5 mM EDTA, 0.3 mg/mL cRNA, 1x PIC and 10 mM PMSF). Specific antibodies (information on S3 Table) were added and nutated for 2 hours at 4°C. After this, 10 μL of protein A agarose beads (Pierce), pre-washed 3 times with 1X IP buffer, were added and nutated 2 more hours at 4°C. Beads were washed 5 times with 1X IP buffer, and RNA-protein complexes were eluted in 125 μL of G-50 buffer containing 0.1mg/mL proteinase K (Sigma) at 37° for 30 minutes. RNA was PCA and chloroform extracted and ethanol precipitated.

### ALYREF overexpression

293i ORF57 and 293i ΔORF57 cells were generated by infecting HEK293 cells with virus made from iSLK-WT or iSLK-ΔORF57 cells complemented in *trans* with an ORF57 expression plasmid. Infected cells were selected with 300 μg/mL of hygromycin.

For siRNA knockdown, we transfected the specified siRNAs as described for iSLK cells. Forty eight hours later, we transfected cells. To do so, 293i ΔORF57 cells grown in a single well of a 6-well plate were transfected with 2 μg of DNA, either 1.6 μg of pcDNA3 + 0.4 μg of ORF50 (to induce lytic reactivation) or 1.6 μg of FLAG-ALYREF + 0.4 μg ORF50 using TransIT-293 reagent following manufacturer’s protocol. Similarly, 293i ORF57 cells were transfected with either 1.6 μg of pcDNA3 + 0.4 μg of ORF50. Thirty-six hours post transfection, total RNA was harvested using TRI reagent. The resulting RNA was then analyzed by qRT-PCR.

## Supporting information

S1 Fig Validation of siRNA knockdown in 293A-TOA cells. (A) Quantitative western blots showing PABPN1, hMTR4 (arrowhead), ARS2 and transient transfected ORF57 protein levels following PAPα/γ, PABPN1, ZFC3H1, hMTR4, ARS2, RBM7 or ZCCHC8 knock down. Actin serves as loading control. (*) unspecific band. (B) Knock-down efficiency of PAPα, PAPγ, ZFC3H1, RBM7 and ZCCHC8 was determined by qRT PCR due to the lack of robust antibodies. Bar graphs showing results from qRT-PCR of PAPα, PAPγ ZFC3H1, RBM7 and ZCCHC8 obtained from 293A-TOA cells where PAPα, PAPγ, ZFC3H1, RBM7 and ZCCHC8 were depleted using siRNAs. Values were normalized to β-actin and plotted relative to siCtrl. (C-D) Because RNA knockdown does not necessarily correlate with protein loss, we assayed for loss of functional activity. To do so, we measured the RNA levels of two known PPD targets (SNHG4 and SNHG19) and two known NEXT complex targets (proEXT1 and proMGST3) by qRT-PCR. (C) Bar graphs showing results of SNHG4 (white) and SNHG19 (black) obtained from 293A-TOA cells where ZFC3H1 was depleted or PAPα and PAPγ were co-depleted. (D) Bar graphs showing results of PROMPTs EXT1 (black) and MGST3 (white) obtained from 293A-TOA cells where RBM7 or ZCCHC8 were depleted. All values are average and the error bars are standard deviations (n = 3).
(TIF)

S2 Fig Pulse-chase assay after RBM7 depletion. (A) Representative northern blot of transcription pulse-chase assay in cells expressing an empty vector or ORF57 and transfected with a two-siRNA pool targeting RBM7. 7SK serves as loading control. (B) Decay curves of biological replicates of the transcription pulse-chase assays after siRBM7; each point is a mean value with standard deviation (n = 3). See Fig 1 for additional details.
(TIF)

S3 Fig Knock-down efficiencies in iSLK-WT and iSLK-ΔORF57 cells. (A-B) Quantitative western blots showing PABPN1, hMTR4 (arrowhead), ARS2 and ORF57 protein levels following PAPα/γ, PABPN1, ZFC3H1, hMTR4, ARS2, RBM7 or ZCCHC8 knockdown in iSLK-WT (A) and iSLK-ΔORF57 cells (B). Actin serves as a loading control; (*) unspecific band. (C) Bar graphs showing results from qRT-PCR of PAPα, PAPγ, ZFC3H1, RBM7 and ZCCHC8 obtained from iSLK-WT (green) and iSLK-ΔORF57 (black) cells where PAPα, PAPγ, ZFC3H1, RBM7 and ZCCHC8 were depleted using siRNAs. (D-G) See S1 Fig for rationale used to determine functional depletion of PAPα/γ, ZFC3H1, RBM7 and ZCCHC8. Bar graphs showing results of PROMPTs EXT1 (D) and MGST3 (E) obtained from iSLK-WT (green) and iSLK-ΔORF57 (black) cells where RBM7 or ZCCHC8 were depleted. (F-G) Bar graphs showing results of SNHG4 and SNHG19 obtained from iSLK-WT (green) and iSLK-ΔORF57 (black) cells where ZFC3H1 was depleted or PAPα and PAPγ were co-depleted. (H) Bar graphs showing results of ORF8, ORF9 and ORF59 obtained from iSLK-WT (green) and iSLK-ΔORF57 (black) cells treated with control siRNAs and iSLK-ΔORF57 cells depleted of RBM7 (gray) or ZCCHC8 (orange). For all samples, lytic reactivation was induced using dox and NaB, and total RNA was harvested 24 hours post lytic induction. For panels C-G, Values were first normalized to β-actin and are shown relative to siCtrl in the same cell line. For panel H, values were calculated relative to the iSLK-WT siCtrl samples. All values are average and the error bars are standard deviations (n = 3).
(TIF)

S4 Fig Validation of immunoprecipitation of proteins for native RIP and UV CLIP. (A-C) Western blot of protein from native RIP of ARS2 (A), ORF57 (B) and hMTR4 (C). (D-E) Western blot of protein from CLIP of ORF57 (D) and hMTR4 (arrowhead) (E). (*) unspecific band. All samples were collected 24 hours post lytic reactivation of iSLK-WT and iSLK-ΔORF57 cells.
(TIF)

S5 Fig Validation of ALYREF overexpression and knock-down efficiency in 293i cells. (A) Western blot of proteins from ALYREF overexpression in 293iΔORF57 cells and knock-down efficiency of ARS2 and hMTR4 (arrowhead). All samples were collected 36 hours following ORF50 transfection to induce lytic reactivation. (*) unspecific band. (B) Bar graphs showing results from qRT-PCR of PAPα (black), PAPγ (white) and SNHG19 (gray) where PAPα and PAPγ were depleted using siRNAs. Values were normalized to β-actin and plotted relative to siCtrl. All values are average and the error bars are standard deviations (n = 3). See Fig 6 legend for experimental details.
(TIF)

S1 Table siRNAs used in this study. All siRNAs were purchased commercially (Silencer Select, Ambion).
(XLSX)

S2 Table Northern blot probe primers and templates. All templates for in vitro transcription were generated by PCR with a T7 promoter on the reverse primer. The one exception was the ORF47 probe which was made by a cut plasmid as indicated.
(XLSX)

S3 Table Antibodies used in this study. All antibodies were commercially available as indicated.
(XLSX)

S4 Table Primers used in this study. Target, sequence, and primer number (ID) for all PCR primers used herein.
(XLSX)
