# Supplementary figures and images for: Kaposi’s sarcoma-associated herpesvirus ORF57 protein protects viral transcripts from specific nuclear RNA decay pathways by preventing hMTR4 recruitment
Source: PLoS Pathog. 2019 Feb 20;15(2):e1007596. doi: 10.1371/journal.ppat.1007596 (PMC6398867; doi:10.1371/journal.ppat.1007596)

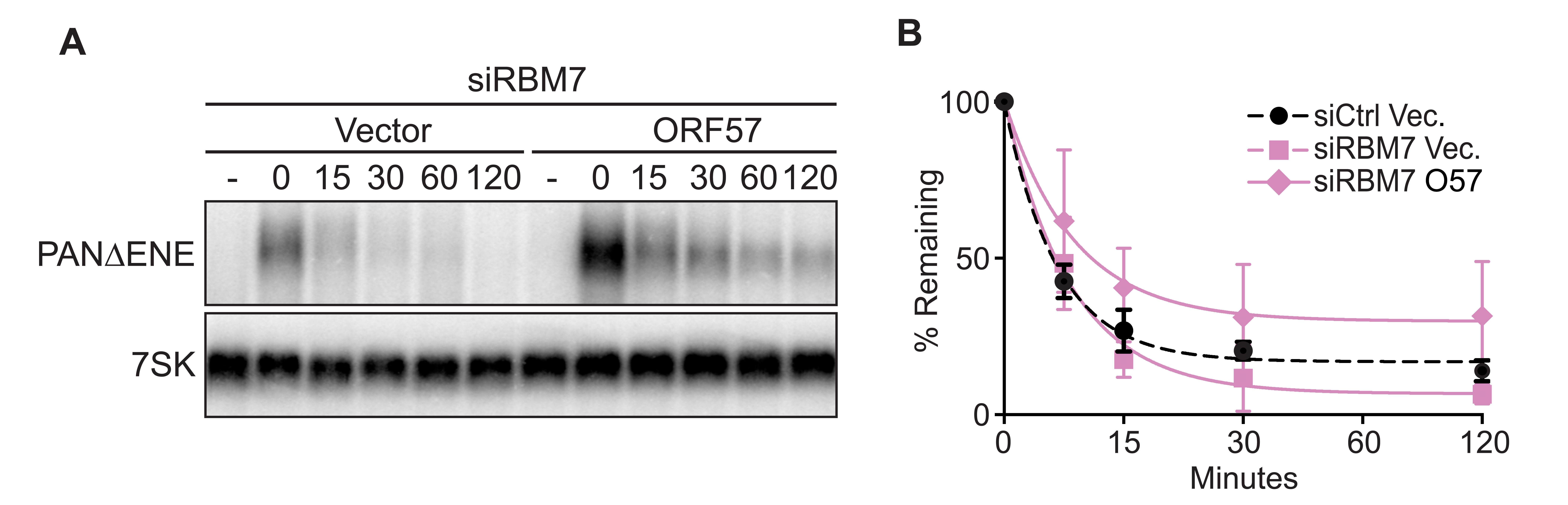

Supplement: S2 Fig — (A) Representative northern blot of transcription pulse-chase assay in cells expressing an empty vector or ORF57 and transfected with a two-siRNA pool targeting RBM7. 7SK serves as loading control. (B) Decay curves of biological replicates of the transcription pulse-chase assays after siRBM7; each point is a mean value with standard deviation (n = 3). See Fig 1 for additional details. (TIF) [file ppat.1007596.s002.tif]

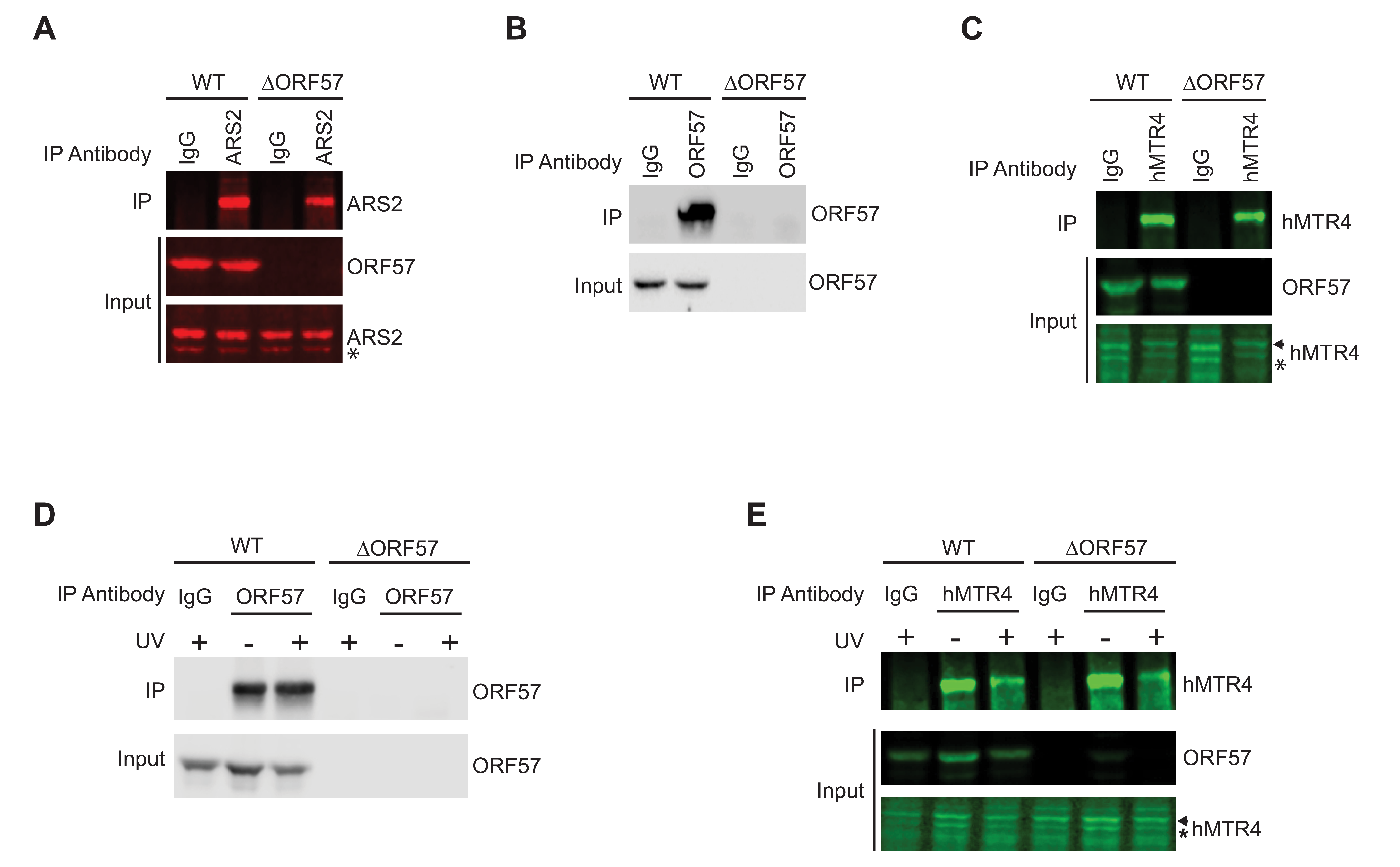

Supplement: S4 Fig — (A-C) Western blot of protein from native RIP of ARS2 (A), ORF57 (B) and hMTR4 (C). (D-E) Western blot of protein from CLIP of ORF57 (D) and hMTR4 (arrowhead) (E). (*) unspecific band. All samples were collected 24 hours post lytic reactivation of iSLK-WT and iSLK-ΔORF57 cells. (TIF) [file ppat.1007596.s004.tif]

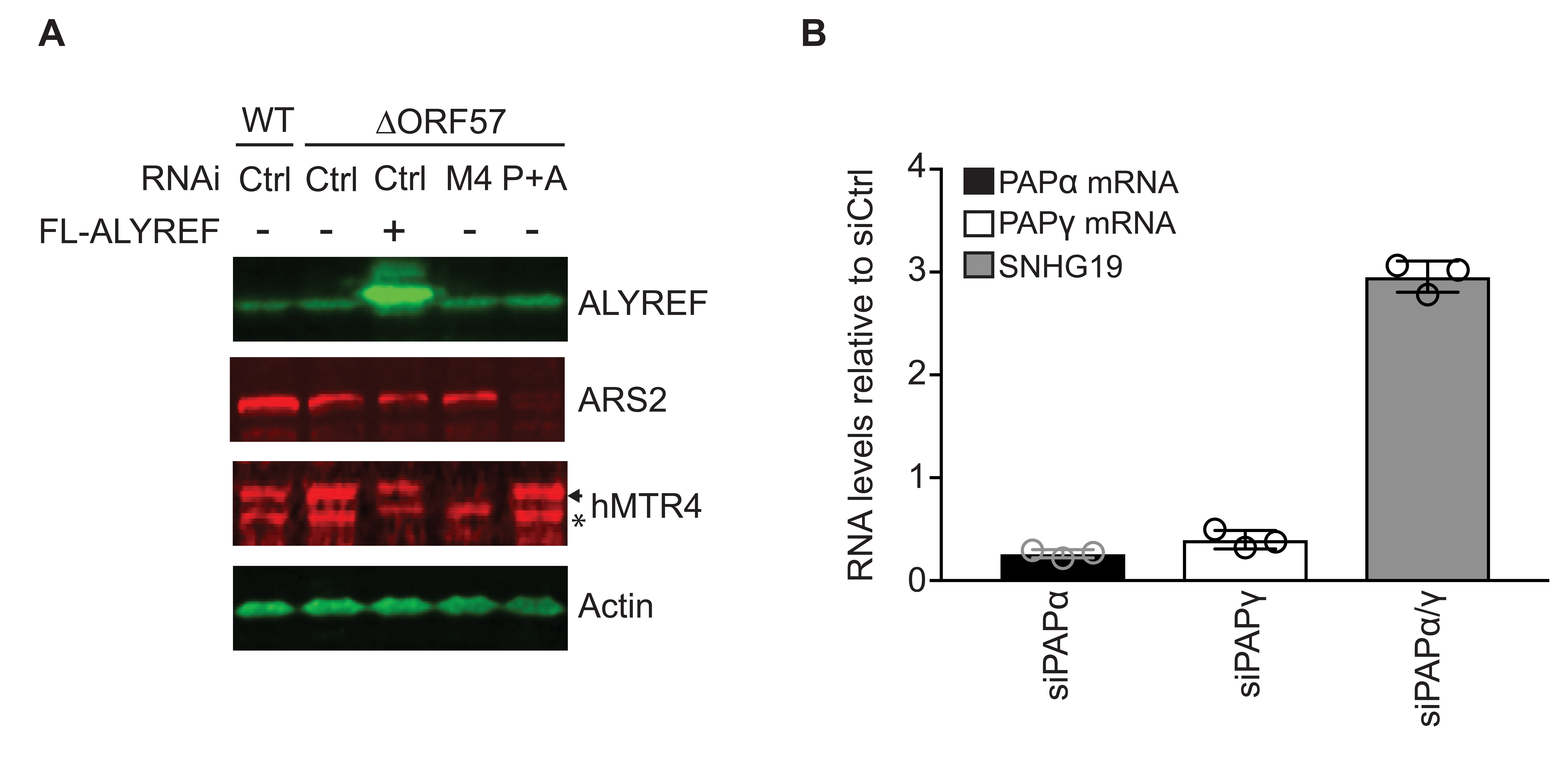

Supplement: S5 Fig — (A) Western blot of proteins from ALYREF overexpression in 293iΔORF57 cells and knock-down efficiency of ARS2 and hMTR4 (arrowhead). All samples were collected 36 hours following ORF50 transfection to induce lytic reactivation. (*) unspecific band. (B) Bar graphs showing results from qRT-PCR of PAPα (black), PAPγ (white) and SNHG19 (gray) where PAPα and PAPγ were depleted using siRNAs. Values were normalized to β-actin and plotted relative to siCtrl. All values are average and the error bars are standard deviations (n = 3). See Fig 6 legend for experimental details. (TIF) [file ppat.1007596.s005.tif]
